# Nutritional Regulation of Mammary Tumor Microenvironment

**DOI:** 10.3389/fcell.2022.803280

**Published:** 2022-02-02

**Authors:** Nikita Thakkar, Ye Bin Shin, Hoon-Ki Sung

**Affiliations:** ^1^ Translational Medicine Program, The Hospital for Sick Children, Toronto, ON, Canada; ^2^ Department of Laboratory Medicine and Pathobiology, University of Toronto, Toronto, ON, Canada

**Keywords:** mammary gland, breast cancer, obesity, white adipose tissue, fasting, tumor microenvironment

## Abstract

The mammary gland is a heterogeneous organ comprising of immune cells, surrounding adipose stromal cells, vascular cells, mammary epithelial, and cancer stem cells. In response to nutritional stimuli, dynamic interactions amongst these cell populations can be modulated, consequently leading to an alteration of the glandular function, physiology, and ultimately disease pathogenesis. For example, obesity, a chronic over-nutritional condition, is known to disrupt homeostasis within the mammary gland and increase risk of breast cancer development. In contrast, emerging evidence has demonstrated that fasting or caloric restriction can negatively impact mammary tumorigenesis. However, how fasting induces phenotypic and functional population differences in the mammary microenvironment is not well understood. In this review, we will provide a detailed overview on the effect of nutritional conditions (i.e., overnutrition or fasting) on the mammary gland microenvironment and its impact on mammary tumor progression.

## Introduction

The relationship between nutrition and cancer has been well established with obesity increasing the risk and progression of breast cancer by 20–40% in post-menopausal women (Munsell et al., 2014). As the mammary gland is a heterogeneous organ constituted of white adipose tissue (WAT), nutritional excess conditions can drive interactions between several populations within the WAT, altering mammary gland integrity and function. In particular, obesity-driven WAT remodeling is known to alter secretory immune and fibrotic profiles (Revelo et al., 2014; Ruiz-Ojeda et al., 2019). Due to obesity, adipocyte size increases beyond the physiological range. This induces cellular stress and apoptosis of adipocytes. As a result, pro-inflammatory macrophages infiltrate to surround these apoptotic adipocytes while extracellular matrix (ECM) is deposited, resulting in a constitutive state of fibro-inflammation within the tissue (Castoldi et al., 2015; Verma et al., 2020). Intriguingly, fibro-inflammation is a hallmark of dysfunctional WAT and is associated with increased cancer incidence (Divella et al., 2016). Obesity-induced mammary WAT fibro-inflammation and interactions of various cell populations provide a microenvironment that contributes to the survival of cancerous cells (Quail and Dannenberg, 2019). Consequently, chronic overnutrition conditions can promote tumor initiation, progression, and metastasis (Park et al., 2000; Ramamonjisoa and Ackerstaff, 2017; Quail and Dannenberg, 2019). Thus, modulation of the mammary WAT microenvironment through nutritional alterations could be an innovative intervention for the prevention and treatment of breast cancer.

Over the past few years, fasting regimens have emerged as effective nutritional interventions to aid with weight loss and improve whole-body metabolism (Varady et al., 2008). While caloric restriction (CR) refers to a restriction in caloric intake, intermittent fasting (IF) is characterized by periodic cycles of fasting followed by free eating which can encompass various nutritional regimes and eating strategies (Klempel et al., 2013; Catterson et al., 2018; Kim et al., 2019). For example, time-restricted feeding (TRF) refers to the restriction of energy intake during specific time frames throughout the day (e.g., 16:8 TRF—16-h of fasting with an 8-h of eating window) (Moro et al., 2016). Other forms of IF include fasting days throughout the week (e.g., 2:1 IF—2 days of normal eating followed by 1 day of fasting) (Kim et al., 2017; Kim et al., 2019; Lee et al., 2020). Fasting regimens protect hematopoietic stem cells from chemotherapy-induced immunosuppression (Cheng et al., 2014), restore healthier cardiometabolic profiles (Almeneessier et al., 2018), improve glucose homeostasis and insulin intolerance (Cho et al., 2019), and initiate an anti-inflammatory response (Kim et al., 2017). Interestingly, emerging evidence has demonstrated that fasting can also negatively impact tumorigenesis (Nencioni et al., 2018).

A longer nighttime fast is associated with improved glycemic control (Lee et al., 2020), decreased breast cancer biomarkers such as leptin and insulin-like growth factor 1 (IGF-1) (Marinac et al., 2015a; Marinac et al., 2015b), and overall reduced breast cancer recurrence (Marinac et al., 2016). In one study, mice were subjected to TRF using high-fat diet (HFD) to induce obesity. These obese mice treated with TRF displayed delayed mammary tumor onset, reduced tumor growth, and total tumor weight (Sundaram and Yan, 2018). In addition to the impact fasting has on breast cancer biomarkers and tumor progression, recent literature has highlighted the therapeutic potential of fasting (Buono and Longo, 2018; Nencioni et al., 2018; Zhao et al., 2021). Fasting prior to administration of chemotherapy (Lee et al., 2012), immune checkpoint blockade therapy (Ajona et al., 2020), and radiotherapy (De La Cruz Bonilla et al., 2019) has shown to decrease tumor growth and improve overall survival, indicating that fasting can improve therapeutic effectiveness in various cancer treatments. As such, fasting regimes are emerging as a promising therapy for cancer patients. However, how fasting modulates the tumor microenvironment to improve cancer outcome is not well understood.

As the mammary gland is composed of WAT, WAT contributes many populations that form the tumor microenvironment. WAT is a heterogeneous tissue composed of adipose stromal cells (ASC), various immune cells, vascular cells, and mammary epithelial cells (Verma et al., 2020). Nutritional excess conditions can alter these microenvironmental populations to favor mammary tumor growth. Alterations in ASC regulate adipocyte expansion, inflammation, and ECM formation (Zhang et al., 2019; Verma et al., 2020). Thereby, ASC play a critical role in maintaining tissue integrity (Hillers et al., 2018). Modulation of immune cells can alter their ability to target abnormal cell growth and control immunosuppression (Cheng et al., 2014). On the other hand, vascular cells regulate nutrient and oxygen supply, which are needed for cancer growth (Yang et al., 2018). Together, these populations have the potential to create a pro-tumor microenvironment which enhances cancer stem cell activity and transforms mammary epithelial cells to promote cancer cell growth (Chamberlin et al., 2017). Hence, as WAT dynamically remodels in response to excess nutrition, these microenvironment populations also remodel to form the tumor microenvironment in breast tissue. As such, investigating the impact of fasting on the mammary tumor microenvironment has been of key interest. In this review, we will summarize the impact of obesity and fasting in the mammary WAT microenvironment cell populations including ASCs, immune cells, vascular cells, mammary epithelial and cancer stem cells, with an emphasis on their contributions to tumorigenesis. For the purposes of this review, studies discussed regarding mammary gland in preclinical models are from inguinal WAT.

## Adipose Stromal Cells and Cancer Associated Fibroblasts

Nutritional excess conditions (e.g., obesity) can disrupt the homeostasis of the mammary gland. Pathological obesity is characterized by the expansion of adipocytes through hyperplastic (increase in number) and hypertrophic (increase in size) growth. ASC largely regulate adipocyte expansion. Also known as mesenchymal stem cells or adipocyte precursor cells, ASC are immunomodulatory bipotential stem cells that have the ability to differentiate into adipocytes or fibroblasts (Casteilla et al., 2011). Their adipogenic differentiation and determination is heavily dependent on PPARγ and C/EBPα, two major early key regulators of adipogenesis (Lechner et al., 2013). However, nutritional conditions can impact their differentiation potential. Obese conditions create a chronic-low grade inflammatory state due to the upregulation of pro-inflammatory mediators such as TNFα (Tzanavari et al., 2010). Upregulation of pro-inflammatory TNFα mediates the inhibition and reversal of adipocyte differentiation by suppressing the expression of adipogenic marker PPARγ, resulting in reduced *de novo* adipogenesis (Karagiannides et al., 2006; Tchkonia et al., 2007). As such, the body stores excess energy through adipocyte hypertrophy. These larger adipocytes exhibit necrotic-like abnormalities and inflammation, contributing to unfavorable fat expansion and insulin resistance (Shao et al., 2018). Furthermore, activation of receptors responsible for immune cytokine signaling such as toll-like receptor 4 exhibit increased ECM deposition and fibrosis (Vila et al., 2014), in part by downregulation of adipogenic marker PPARγ (Wei et al., 2010). Hence, coupled with adipocyte expansion, the microenvironment of obese WAT experiences hypoxia, adipocyte death, dysregulated angiogenesis, ECM deposition, and immune cell infiltration (Verma et al., 2020), overall creating an unhealthy microenvironment. Consequently, this unhealthy microenvironment can inhibit ductal growth, impair mammary gland function, and foster tumor growth (Lin and Li, 2007; Ullah et al., 2017). Thus, healthy WAT expansion is of vast interest to prevent the formation of a pro-tumor microenvironment.

Over recent years, fasting has been shown to be effective against obesity largely due to its effect on the adipocyte population. Obese mice subjected to 16 weeks of an IF regimen consisting of 2 feeding days followed by 1 fasting day exhibited decreased WAT weight as well as adipocyte size, without major alterations in whole-body lean mass (Kim et al., 2017). Tang et al. illustrated that adipogenesis is enhanced in mice subjected to 24-h refeeding after a 72-h fast through an upregulation of differentiation transcription factors promoting adipogenesis such as PPARγ and C/EBPα (Tang et al., 2017). In addition, a hallmark of fasting-induced WAT changes includes *beiging* or *browning*, a phenomenon describing adipocytes that acquire brown adipocyte-like characteristics. Subjecting mice to 40% CR, 40% of normal caloric intake, diet for 5 weeks resulted in greater hyperplastic growth, reduced hypertrophy, and multilocular lipids, indicative of WAT *browning* (Fabbiano et al., 2016). Furthermore, inguinal mammary WAT exhibited increased expression of *browning* markers such as *UCP1, Cidea, PPARγ*, and adipose-fatty acid binding protein 4 (*Fabp4*) (Fabbiano et al., 2016). Similarly, alternate-day fasting for 15 cycles resulted in increased expression of thermogenic *browning* markers and multilocular lipid accumulation of inguinal WAT. This WAT *browning* was demonstrated in part due to elevated levels of acetate and lactate (Li et al., 2017). Since WAT *browning* promotes healthy storage of fat through hyperplastic growth (Fabbiano et al., 2016), IF-induced *browning* serves as a potential therapy to promote healthy adipogenesis, maintain tissue integrity, and combat the growing obesity epidemic.

While WAT *browning* has been established as an essential component mediating fasting-induced metabolic benefits, recent evidence has highlighted novel interactions for resident beige adipocytes in mammary tumors. To investigate the influence of UCP1+ beige adipocytes on tumor growth, Singh et al. sorted UCP1+ and UCP1- fractions from xenografted breast cancer tumors. Subcutaneously injecting mammary tumor fraction depleted for UCP1+ adipocytes into nude mice significantly reduced tumor growth, suggesting that beige adipocytes may contribute to breast cancer development (Singh et al., 2016). Additionally, membrane-bound extracellular vesicle exosome interactions between adipocytes and tumor cells have emerged as a potential mechanism to induce WAT *browning* and lipolysis in the mammary tumor (Zhao et al., 2018; Wu et al., 2019). Co-culturing breast cancer cells and mature adipocytes decreases lipid droplet size, number, triglyceride content, and adipogenic marker *PPARγ,* while increasing *UCP-1* levels and lipolytic activity. Subjecting cancer cells to cultured medium from mature adipocytes increases invasion and downregulation of epithelial marker E-cadherin (Wu et al., 2019), indicating increased aggressiveness. These studies suggest that tumor cells can take advantage of surrounding adipocytes (cancer-associated adipocytes) to induce thermogenesis and lipolysis to amplify their nutrient supply. Often upregulated by lipolysis and obesity is *Fabp4*. Genetic deletion of *Fabp4* eradicated obesity-associated mammary tumor growth and development (Hao et al., 2018), highlighting a potential biomarker to predict obesity-induced aggressiveness. As fasting has been shown to induce WAT *browning* (Kim et al., 2017), the impact fasting has on preventing or exacerbating cancer-associated adipocytes in the mammary tumor is inconclusive. Since adipocytes within the mammary gland supply lipids to neighboring cells for nutritional functions (Zwick et al., 2018), it is essential to understand whether fasting-induced lipolytic activity could increase energy supply for cancer cells and potentially aggravate progression of mammary tumors.

As adipocytes surrounding the tumor lose lipids, these cells can acquire fibroblast-like features. Thereby, cancer-associated adipocytes can represent an intermediate population with fibroblast features including increased fibronectin, collagen 1, fibroblast-stromal protein (FSP) (Bochet et al., 2013), alpha-smooth actin (αSMA) (Inoue et al., 2016), fibroblast activation protein (FAP) (Kahounova et al., 2018), and vimentin expression (Hsia et al., 2016). Although the origin is unknown, one attractive theory is that mature adipocytes can transform into cancer-associated adipocytes and acquire fibroblast features to become fibroblast-like cells (Bochet et al., 2013). These fibroblast-like ASCs are known as cancer associated fibroblasts (CAF). Activated CAFs can impact tumor microenvironment and modulate cancer metastasis through ECM remodeling, angiogenesis, and growth factor secretion (Sahai et al., 2020). Isolating circulating ASCs from obese donors and subjecting them to breast cancer MCF-7 cells *in vitro* demonstrated decreased adipogenic differentiation potential and increased expression of fibrotic markers such as αSMA, FAP, and FSP (Strong et al., 2017). Furthermore, examination of ASC isolated from diet-induced obese mice mammary gland WAT revealed decreased differentiation and upregulation of activated fibroblasts marker, αSMA. Co-inoculating obese mice ASCs and mammary tumor cells into lean mice resulted in larger tumor growth, enhanced proliferation, and increased invasion (Hillers et al., 2018). In addition, an independent study demonstrated that in lung cancer, CAFs promote epithelial-to-mesenchymal transition and enhance the metastatic potential of cancer cells through an IL-6 mediated pathway. Administering IL-6 neutralizing antibody abolished the effects of CAF-induced cell migration, possibly leading to reduced metastasis (Wang et al., 2017). These findings suggest that HFD-induced obesity can reduce adipogenic differentiation of ASCs while augmenting ASC conversion to CAF (Hillers et al., 2018).

A study by Wu et al. demonstrated that mature adipocytes expressing programmed death-ligand 1 (PD-L1) inhibit activation of anti-tumor CD8+ T cells when administered anti-PD-L1 antibody *in vitro*. While depletion of PD-L1 expression on mature adipocytes promotes CD8+ T cell activation, ASC specific PD-L1 expression inactivates cytotoxicity of CD8+ T cell, suggesting context dependent immune modulatory function of PD-L1. Pharmacologic inhibition of adipogenesis in mammary tumors reduces PD-L1 expression and enhances anti-tumor efficacy, highlighting a potential role of the ASC population to modulate therapeutic effectiveness by controlling adipogenesis (Wu et al., 2018). Currently, there are no published studies showing the direct impact fasting has on the ASC or CAF population in breast cancer. As fasting is known to decrease IL-6 (Speaker et al., 2016) and is thought to modulate the ASC population to promote healthy adipocyte expansion, understanding how the ASC and CAF populations are modulated in response to fasting is critical to expand our knowledge into fasting-induced microenvironmental changes.

## Immune Cells—Myeloid Cells

Obesity induces mammary WAT expansion and is coupled with an accumulation of immune cells, specifically macrophages (Kolb and Zhang, 2020; Colleluori et al., 2021). WAT-resident macrophages proliferate while newly recruited monocyte-derived macrophages accumulate. These macrophages can assist with regulating physiological processes and maintaining metabolic function of WAT. In particular, these infiltrated pro-inflammatory adipose-tissue macrophage (ATM) surround necrotic cells to reabsorb lipids forming crown-like structures (CLS). As a highly abundant population, CLS macrophages secrete several pro-inflammatory mediators including TNFα, IL-6, CRP, and MCP-1 (Lumeng et al., 2007; Coats et al., 2017) into the microenvironment, resulting in chronic low-grade inflammation. Since WAT constitutes a major component of the breast tissue microenvironment, this chronic low-grade inflammation is associated with increased breast cancer risk, reduced overall survival, and recurrence-free survival (Pajares et al., 2013; Ecker et al., 2019).

Historically in WAT tissue biology, there are two distinct macrophage population. M1-like CD11c + pro-inflammatory macrophages are induced by pro-inflammatory factors such as interferon gamma (IFN γ ), IL-6, IL-1β, and predominantly contribute to the formation of CLS. In comparison, classical CD206+ M2-like macrophages are anti-inflammatory, contributing to tissue repair and production of anti-inflammatory cytokines such as IL-4 and IL-13 (Novak and Koh, 2013). In general, to maintain tissue integrity and homeostasis of WAT, a balance between pro-inflammatory and anti-inflammatory macrophages is required (Lumeng et al., 2007). Intriguingly, as obesity disturbs this balance by favoring M1-like macrophages, IF has been shown to restore balance with M2-like polarization (Zhao et al., 2018). Kim et al. demonstrated that induction of VEGF through a 2:1 feeding-to fasting-regimen of IF induces alternative activation of M2-like macrophages, promoting visceral WAT *browning* and thermogenesis (Kim et al., 2017). Similarly, 40% CR stimulates *browning* of mammary inguinal WAT through increased eosinophil infiltration, type 2 cytokine signaling and M2-like macrophage polarization. Suppression of type 2 signaling prevented *browning* and mammary inguinal WAT loss with CR, highlighting the importance of immune signaling and macrophage polarization for CR benefits (Fabbiano et al., 2016). Since chronic low-grade inflammation and presence of M1-like CLS are associated with increased risk of tumor onset (Carter et al., 2018; Faria et al., 2020), the polarization of M2-like macrophages through IF or CR could contribute to restoring a balance between M1-like and M2-like macrophages in the mammary microenvironment. This subsequently could decrease mammary WAT inflammation and potentially suppress the initiation of mammary tumor formation.

The stage at which IF induces M2-like macrophage polarization and accumulation could alter the benefits of IF on mammary tumorigenesis. Whereas M2-like macrophage accumulation prior to tumor onset can decrease inflammation and thereby suppress tumor onset, accumulation of M2-like macrophages during tumor progression may worsen prognosis. Jeong et al. established an association between M1-like macrophages and higher overall and disease-free survival in tissue microarrays of human invasive breast cancer, suggesting M2-like macrophages accelerate tumor progression (Jeong et al., 2019). Thus, further research is necessary to understand the impact IF-induced M2-like polarization could have during different stages of tumor development. As extremely plastic cells, increasing literature indeed highlights many other macrophage subcategories (Chavez-Galan et al., 2015). In the tumor context, using classical M1-like and M2-like macrophages is insufficient to characterize the tumor-associated macrophage population. Understanding the various roles different tumor-associated macrophage populations play in mammary tumor development and how these populations are altered by IF, could provide novel insight into the shaping of the tumor microenvironment (Narita et al., 2018; Mao et al., 2021).

Though macrophages are the most abundant leukocytes, eosinophils and myeloid derived suppressor cells (MDSC) also exist in the mammary gland. As shown by Fabbino et al., CR stimulates *browning* of mammary WAT through increased eosinophil infiltration (Fabbiano et al., 2016). Increased serum eosinophils counts are associated with better breast cancer prognosis, response to therapy, and long-term survival (Martens et al., 2016). Notably, a positive association between eosinophilia and disease outcome has been detected widely in metastatic melanoma (Simon et al., 2020) and recently in breast cancer (Onesti et al., 2020). In fact, Zheng et al. demonstrated that immune checkpoint blockade administration of anti-CTLA-4 treatment in MMTV-Polyomavirus Middle T-antigen (MMTV-PyMT) model increased eosinophil infiltration. Pharmacological depletion of eosinophils decreased the anti-tumor effect of CTLA-4, thereby promoting tumor growth (Zheng et al., 2020). As eosinophils counts are predictive for cancer prognosis (Martens et al., 2016) and play at least a partial role in mediating immune checkpoint blockade antibody response in mammary tumors (Zheng et al., 2020), IF-mediated upregulation in eosinophils may contribute to enhancing therapeutic efficacy and consequently improving breast cancer prognosis.

On the other hand, little is known about the direct impact of fasting on the MDSC population in the mammary gland and tumor microenvironment. Under HFD conditions, mammary tumors isolated from obese mice displayed increased tumor progression and enhancement of the MDSC population. Depleting MDSC in obese mice protected against diet-induced metabolic dysfunction and inflammation, which was sufficient to decrease tumor volume, liver metastasis, and improve overall survival. They discovered that HFD induces MDSC′ PD-L1 expression, thereby inactivating CD8+ T cell cytotoxic activity (Clements et al., 2018) and enhancing immunosuppression. However, the direct interaction of fasting on the immunophenotype of MDSC has not been clearly documented.

Intriguingly, metformin, an AMP-activated protein kinase (AMPK) activator, has become an emerging therapy in breast cancer (Goodman et al., 2014). Preclinical AMPK activator, OSU-53, suppresses breast cancer (MDA-MB-231 xenograft) growth by 47–49% (Lee et al., 2011)**.** Importantly, AMPK and its downstream pathways have been shown to be activated by IF or fasting (Kajita et al., 2008; Viscarra et al., 2011). Since obesity-induced chronic low-grade inflammation is known to increase tumor-associated MDSCs, anti-obesity interventions such as IF could modulate the MDSC population (Salminen et al., 2019). In a study by Turbitt et al., mice were subjected to one of three possible treatments including 10%kcal HFD feeding, 60%kcal HFD feeding, or 30%kcal CR feeding for 16 weeks. These dietary regimens produced overweight, obese, and lean mice respectively which were subsequently injected with pancreatic tumor cells. A linear association between greater adiposity and tumor growth was observed, with obese animals bearing the largest tumors. Additionally, overweight/obese tumors contained a lower CD8: MDSC ratio, with an overall greater proportion of MDSC’s and a lower proportion of CD8+ T cell (Turbitt et al., 2019). Though this study was conducted in pancreatic cancer, systemic activation of AMPK through CR could have important implications in modulating the MDSC population in breast cancer.

## Immune Cells—Lymphoid Cells

While myeloid populations like tumor-associated macrophages, eosinophils, and MDSCs are predominantly involved in immunosuppression, lymphoid populations inherently aid with alleviating this immunosuppression and participating in tumor-killing cytotoxic activity. In particular, CD8+ T cells play a critical role in modulating the mammary WAT microenvironment in response to obesity and tumorigenesis. MMTV-PyMT mice fed 60% HFD for 14–20 weeks promoted tumor initiation and progression via modulation of CD8+ T cell population (Kado et al., 2019). Obesity induced a phenotypic switch in tumor CD8+ T cells to promote early exhaustion as cells acquire exhaustive immune checkpoint receptor PD-1 (Kado et al., 2019). Consequently, the increase in PD-1+ CD8+ cells by diet-induced obesity resulted in reduced expression of cytotoxic genes such as IFN γ and granzyme B (GzB) (Kado et al., 2019), suggesting that obesity decreases anti-tumor cytotoxic activity by induction of T cell exhaustion. Along with decreased proliferative capacity and activity of CD8+ T cells, an independent study discovered HFD initiates metabolic reprogramming of cancer cell to increase lipid uptake, while starving CD8+ T cells (Ringel et al., 2020). Prolyl-hydroxylase 3 (PHD3) is a protein in normal cells that has been shown to inhibit excessive lipid metabolism. Overexpression of PHD3 in tumor cells enhanced anti-tumor activity by blocking cancer cell metabolic reprogramming, resulting in slower tumor growth (Ringel et al., 2020). As similar metabolic reprogramming was observed in human cancers, this study provided a novel insight into changes in cellular components of the tumor microenvironment in response to diet-induced obesity. Taken together, these studies highlight the immunosuppressive nature of diet-induced obesity on tumor-associated CD8+ T cells.

Intriguingly, Biase et al. revealed a fasting-mimicking diet (FMD), a high-fat and low-carb CR diet, to enhance CD8+ tumor-infiltrating lymphocytes (TIL) in breast cancer (Di Biase et al., 2016). Triple negative breast cancer 4T1 tumor-bearing female mice subjected to 4 days of FMD sensitized tumors to chemotherapeutic doxorubicin and cyclophosphamide treatment as tumor volume was significantly decreased by 3-fold. The combination of FMD and chemotherapy (doxorubicin) significantly increased CD8+ TILs and their cytotoxic enzyme (e.g., GzB) and enhanced tumor cell apoptosis (Di Biase et al., 2016). Conversely, depleting CD8+ TIL’s by neutralizing antibody increases regulatory T cells (Tregs). Subjecting Balb/C nude mice lacking T-lymphocytes to FMD was not effective at reducing tumor size, increasing GzB, or cleaved-caspase-3 levels, highlighting the critical role that TIL play in mediating FMD effects (Di Biase et al., 2016). This study uncovered Heme-Oxygenase 1 downregulation to be essential for FMD-induced increase in CD8+ TIL cytotoxicity. Overall, this study suggests that FMD increases CD8+ TIL cytotoxicity by reducing Heme-Oxygenase 1 expression with associated Treg downregulation (Di Biase et al., 2016). Since HFD was shown to change metabolic programming of cancer cells in the tumor microenvironment and IF is known to decrease several metabolic parameters such as insulin and glucose, future studies should investigate how IF can impact tumor microenvironment metabolic programming. In particular, it would be interesting to see the impact of IF on PHD3. This could provide promising novel targets and IF mimetics that could improve anti-tumor activity and enhance current therapeutics.

Another key lymphoid population that plays an important role in anti-tumor properties is Natural Killer cells (NK). Though an important population in the tumor context, NK cells have not been thoroughly examined for their role in obesity in the mammary gland (Ohmura et al., 2010). Under obese conditions, alterations in resident NK cells have been observed in epididymal fat but not subcutaneous fat (Lee et al., 2016). A subpopulation of NK cells resembling T cells exists and are referred to as NKT cells. With just 4 days of HFD, NKT cells are activated and promote M2-like macrophage polarization in the depot. NKT-deficient CD1d −/− mice subjected to HFD challenge showed impaired metabolic parameters, without polarization of M2-like macrophages (Ji et al., 2012). This suggests that NKT cells play a WAT depot specific role in contributing to obesity-induced WAT remodeling. Additionally, compared to lean counterparts, obese mammary tumor-bearing mice demonstrated decreased ligand NKp46 expression on circulating NK cells and increased activating NK receptor NKG2D ligand MULT1 expression in visceral WAT (Spielmann et al., 2020). As such, this paper alluded that NK cells may be occupied in managing inflamed visceral WAT microenvironments during obese conditions and therefore are unable to kill mammary tumor cells, leading to accelerated tumor growth. Future research into the effect of prolonged HFD feeding on NK/NKT receptor and ligand activity in the mammary gland could provide insight into whether there is a differential role for NK cells in mammary WAT.

In an independent liver study, mice subjected to a 3 days fast increased tumor-necrosis factor-related apoptosis inducing ligand and CD69+ NK cells, without major alterations in resident NK cell number. These NK cells displayed enhanced antitumor function in comparison to the fed group (Dang et al., 2014). Though this study was conducted in the liver, such an effect in a mammary tumor could be instrumental in targeting tumors. As one of the key populations responsible for cytotoxic activity in tumors, understanding how NK cells change under obese conditions in the mammary gland could shed light into on their potential contribution to anti-tumor activity by fasting regimens.

**FIGURE 1:**
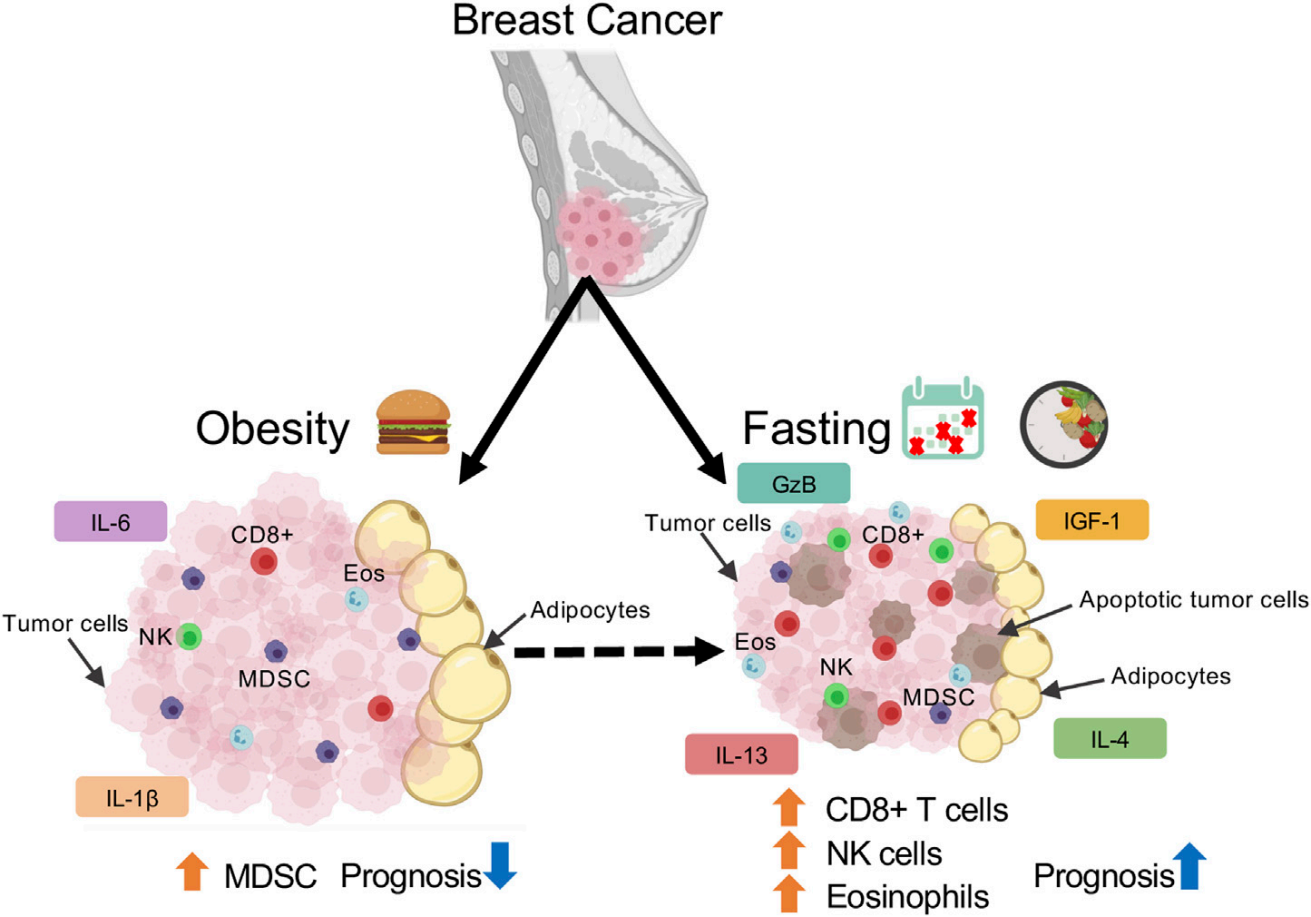
Schematic depicting immune population changes in mammary tumor microenvironment in response to obesity and fasting conditions.

Collectively, fasting regimens can alleviate immunosuppression by potentially increasing eosinophil, CD8+ T cells, and NK cells while decreasing MDSC cells (Figure 1). However, as fasting is a systemic response, it may alter immune populations not only in the mammary gland or tumor, but in circulation as well. Several studies have alluded to variations in cell number, immune cell production, and T cell priming in lymphoid organs upon fasting (Strissel et al., 2010; Shushimita et al., 2014; Buono and Longo, 2019; Nagai et al., 2019; Rangan et al., 2019). Upon 50% CR, CD8+, CD4+ T cells, Tregs, NK, and mature B cells decreased in WAT and spleen, yet CD8+ and CD4+ T cells were increased in bone marrow (Collins et al., 2019). This allows cells to preserve a state of energy conservation and allow for T cell priming by CXCR4-CXCL12 activity on CD4+ memory T cells. As the major function of CD4^+^ memory T cells is immunosurveillance, memory T cell homing was associated with enhanced protection against infections and tumors (Collins et al., 2019). Similarly, another study revealed prolonged fasting (48-h) significantly deceased white blood cell and hemopoietic stem cell numbers. Refeeding after fasting increased the number and activity of hematopoietic progenitor populations, suggesting that refeeding can rejuvenate immune cells to weaken immunosuppression caused by chemotoxicity in a cancer context (Cheng et al., 2014). In a randomized study of 129 patients, subjecting HER2-negative early breast cancer patients to FMD and chemotherapy reduced disease progression and DNA damage in plasma T-lymphocytes (De Groot et al., 2020), thereby reducing hematological toxicity (De Groot et al., 2015). Altogether, these studies imply that fasting accelerates recovery after chemotoxicity in breast cancer through T cell priming and functioning of the bone marrow. Future research investigating changes in lymphoid populations within lymphoid organs, tumor, and in circulation upon fasting and refeeding could provide insight into how fasting changes the lymphoid immune response.

## Vascular Cells

With obesity, several pro-angiogenic factors are secreted from the microenvironment to induce a transient switch to activate angiogenesis. Activation of angiogenesis is critical to ensure a sufficient supply of nutrients and oxygen to cells for healthy expansion. However, a balance between pro-angiogenic and anti-angiogenic factors is necessary to prevent endothelial cell dysfunction (Herold and Kalucka, 2020). Obesity-related expansion of WAT is accompanied by endothelial dysfunction as there is an increase in the pro-angiogenic and pro-inflammatory stimulus. Maintaining an adequate supply while sustaining proper endothelial function is essential to prevent WAT inflammation, fibrosis, and pockets of hypoxia, all of which contribute to unhealthy WAT and are hallmarks for mammary tumorigenesis.

A key pro-angiogenic molecule implicated in mammary gland expansion and tumorigenesis is Vascular Endothelial Growth Factor (VEGF). VEGF is known to regulate vascular permeability, angiogenesis, and the expansion of lymphatic vessels through lymphangiogenesis (Kim et al., 2017). Under basal conditions, overexpression of adipose-specific VEGF triggers angiogenesis and *browning* of inguinal mammary WAT (Elias et al., 2012; Sun et al., 2012; Sung et al., 2013). Intriguingly, transplanting this overexpressing adipose-VEGF tissue into diet-induced obese mice improved systemic metabolic benefits and reduced inflammation (Park et al., 2017)**.** Notably, 24-h fasting significantly increases inguinal WAT-VEGF expression in overall tissue and adipocytes (Hua et al., 2021). In fact, metabolic benefits of IF such as WAT *browning,* and M2-like macrophage polarization have been shown to be mediated by adipose-VEGF expression. Whereas adipose-VEGF knockout mice are unable to gain metabolic benefits of IF, periodic expression of adipose-VEGF (i.e., IF-mimicking effect) is sufficient to induce IF metabolic improvements in non-fasted animals (Kim et al., 2017), highlighting the important role VEGF plays in promoting the healthy remodeling of obese WAT.

As upregulation of VEGF promotes healthy expansion of WAT, this same mechanism can promote growth and dissemination of solid tumors such as breast cancer. Unlike the formation of mature vessels under normal and obese conditions, intratumor vessels are irregular, disorganized, and leaky, leading to hypoxia and inefficient delivery of antitumor agents into tumor microenvironment (Yang et al., 2018). In contrast to obese conditions where it is beneficial to upregulate VEGF, in the tumor context VEGF is thought to aid in the proliferation and expansion of tumor. Silencing VEGF expression via small interfering RNA significantly reduced tumor growth and angiogenesis in breast cancer MCF-7 xenografted mice (Chen et al., 2017). As such, combining traditional chemotherapies with anti-VEGF therapies has been extensively investigated in many cancers. However, anti-VEGF therapy such as bevacizumab has largely failed to improve survival in breast cancer patients. In particular, obese breast cancer patients respond poorly to anti-VEGF therapy due to decreased sensitivity in the tumor (Incio et al., 2018). HFD feeding in breast cancer cell E0771 inoculated mice decreased anti-VEGF therapy efficacy from 50 to 28%. These obese tumors experienced hypovascularity, hypoxia, and increased abundance of cancer-associated adipocytes (Incio et al., 2018). Treatment of anti-VEGF therapy-induced cancer cell necrosis in adipocyte-poor regions while adipocyte-rich regions remained viable, attributed to increased pro-inflammatory molecule IL-6. Inhibiting IL-6 and VEGF increased functional vascular density, reduced hypoxia, attenuated infiltration of Tregs, decreased mammary tumor growth, and metastasis (Incio et al., 2018). Furthermore, combining anti-VEGF blockade with inhibition of IL-6 plus chemotherapeutic agent doxorubicin in obese mice delayed tumor progression similar to lean mice on VEGF blockade and doxorubicin. Inhibition of IL-6 did not further delay progression in lean animals, thereby suggesting that obesity promotes resistance to anti-VEGF therapy in breast cancer specifically by IL-6 (Incio et al., 2018). As subcutaneous IL-6 is known to decrease with fasting (Speaker et al., 2016)**,** combining IF with anti-VEGF therapy could have a beneficial impact on tumor growth. Research examining the impact of IF could prove to be a powerful tool for anti-VEGF therapy effectiveness. As anti-VEGF therapy has shown poor clinical results in mitigating breast cancer, this insight is essential to understand the translatability of IF into a clinical setting.

In addition to VEGF, angiopoietin-like 4 (ANGPTL4) has been investigated in implanted E0071 mammary tumors. Knocking out or neutralizing ANGPTL4 in mice decreased obesity-induced angiogenesis and tumor growth (Kolb et al., 2019). As 24-h fasting in humans has been shown to increase mRNA and protein ANGPTL4 in mammary WAT (Ruppert et al., 2020), fasting may accelerate obesity-induced tumor angiogenesis. As a result, this could contribute to cancer cell survival through an ample supply of oxygen and nutrients. However, De Lorenzo et al. investigated the impact of 4T1 cell implantation into the mammary gland of BALB/c mice after 5 weeks of 40% CR. Along with CR decreasing tumor weight, metastasis, cell proliferation, and increasing apoptosis, CR mice also displayed significantly lower intratumor microvessel density than control counterparts. This significant decrease was attributed to decreased total vessel length and circulating serum VEGF levels, suggesting CR decreases tumor angiogenesis (De Lorenzo et al., 2011). Collectively, there is a lack of studies investigating the impact of IF in the tumor microenvironment on pro-angiogenic, anti-angiogenic factors, and overall vascular cells. As lymphangiogenesis is an integral process through which cancer cells metastasize, understanding the impact IF has on the vascular microenvironment will provide insight into cancer cell dissemination into local and distant organs. In-depth analysis of vascular markers and collective population will provide a better understanding of IF’s potential to modulate the breast cancer vascular microenvironment.

## Mammary Epithelial Cells and Cancer Stem Cells

A predominant population of the mammary gland are mammary epithelial cells. Remodeling of these cells initiates processes that are characteristic to the mammary gland during lactation and puberty (Olson et al., 2010). During these processes, mammary epithelial cells interact closely with neighboring adipocytes (Colleluori et al., 2021). As such, the mammary epithelial population is sensitive to nutritional conditions. Subjecting C57BL/6–60% HFD decreased basal/myoepithelial specific markers while increasing mammary epithelial progenitor activity and estrogen receptor expression, specifically in luminal cells. Interestingly, switching mice fed HFD for 15 weeks to control diet for a further 5 weeks, mimicking a weight loss regimen, reversed the observed epithelial cell changes. These results were recapitulated in human mammary tissue, indicating that obesity can directly alter stem/progenitor epithelial populations (Chamberlin et al., 2017).

To investigate mammary epithelial gland structure alterations in response to obesity, Mustafi et al. subjected 4-week-old spontaneous tumor developing simian virus 40 large T antigen (SV40taG) mice to 60% HFD for 8 weeks. In addition to enhanced tumor progression, *ex vivo* MRI and histology demonstrated denser parenchyma, irregularly enlarged ducts, dilated blood vessels, increased WAT, and increased tumor invasion (Mustafi et al., 2017), showcasing HFD-induced mammary epithelial dysregulation. Indeed, dissociation of obese mammary tumor into single cells grown *in vitro* revealed increased proliferation rates and self-renewal capacity in an independent study. Growing these cancer stem cell-enriched populations on collagen-coated migration chambers showed increased invasiveness, increased expression of the mesenchymal marker N-cadherin, and higher cancer stem cell-associated genes *Sox2* and *Notch Receptor 2* (Hillers-Ziemer et al., 2020). Collectively, these studies suggest amplification of cancer stem cell activity, proliferation, and aggressiveness in response to obesity.

While obese conditions enhance mesenchymal marker expression, CR has importantly been shown to affect epithelial-to-mesenchymal transition in the mammary tumor. As a critical pathway involved in tumor invasion, growth, and metastasis, Dunlap et al. investigated the impact of CR focusing on two characterized cell types in the mammary tumor of transgenic MMTV-WNT-1 mice. Compared to epithelial cells (CD44 high/CD24 high), mesenchymal cells from MMTV-WNT-1(CD44 high/CD24 low) mice were tumor-initiating cells with greater tumorsphere-forming and migration abilities. Obese tumors from mice fed with 60% HFD prior to and after tumor implantation experienced upregulated mesenchymal cells and overall enhanced epithelial-to-mesenchymal transition characterized by markers such as *N-cadherin*, *Fibronectin*, transforming growth factor-β (*TGFβ*), *Snail,* and *Oct4*. On the other hand, 30% CR suppressed tumor progression, inhibited epithelial-to-mesenchymal transition and intratumoral adipocyte accumulation, implying that dietary interventions such as CR can modulate epithelial-mesenchymal-transition thereby affecting the progression of mammary tumors (Dunlap et al., 2012).

In addition to the influence nutritional conditions can have on epithelial cells, adipokines regulated by nutritional conditions such as leptin can impact the mammary gland and tumor microenvironment. Increased under obese conditions, leptin is associated with increased breast cancer risk and thereby serves as a potential biomarker for post-menopausal overweight/obese patients (Pan et al., 2018). Upregulation of leptin disrupts epithelial polarity and sensitizes non-cancer cells to proliferative stimuli to expand the stem cell/progenitor population, subsequently initiating early stages of malignancy (Tenvooren et al., 2019). Additionally, leptin promotes expression of epithelial-to-mesenchymal transcription factors, cancer stem cell activity, expression of metastatic *TGFβ1* pathway (Mishra et al., 2017; Olea-Flores et al., 2019), in part by activation of inflammasomes (Raut et al., 2019). As leptin is secreted by adipocytes within the breast tissue, leptin can alter the tumor microenvironment. Increased leptin due to obesity explains the increased risk of invasive/metastatic tumors and overall poor survival in obese breast cancer patients. Intriguingly, numerous studies have shown fasting significantly decreases leptin (Trepanowski et al., 2018; Al-Rawi et al., 2020) and increases adiponectin (Varady et al., 2010; Kim et al., 2017). Adipose-secreted cytokine adiponectin decreases breast cancer growth by the accumulation and activation of autophagosomes resulting in autophagic cell death in the mammary tumor (Chung et al., 2017). Currently, there lacks research examining the effect of fasting-related decreases in leptin and increases in adiponectin on mammary epithelial polarization, cancer stem cell activity, autophagy, invasion, and metastasis. This could enhance our knowledge and provide a potential explanation for the delayed onset observed in mammary tumor fasting studies.

## Discussion

**FIGURE 2:**
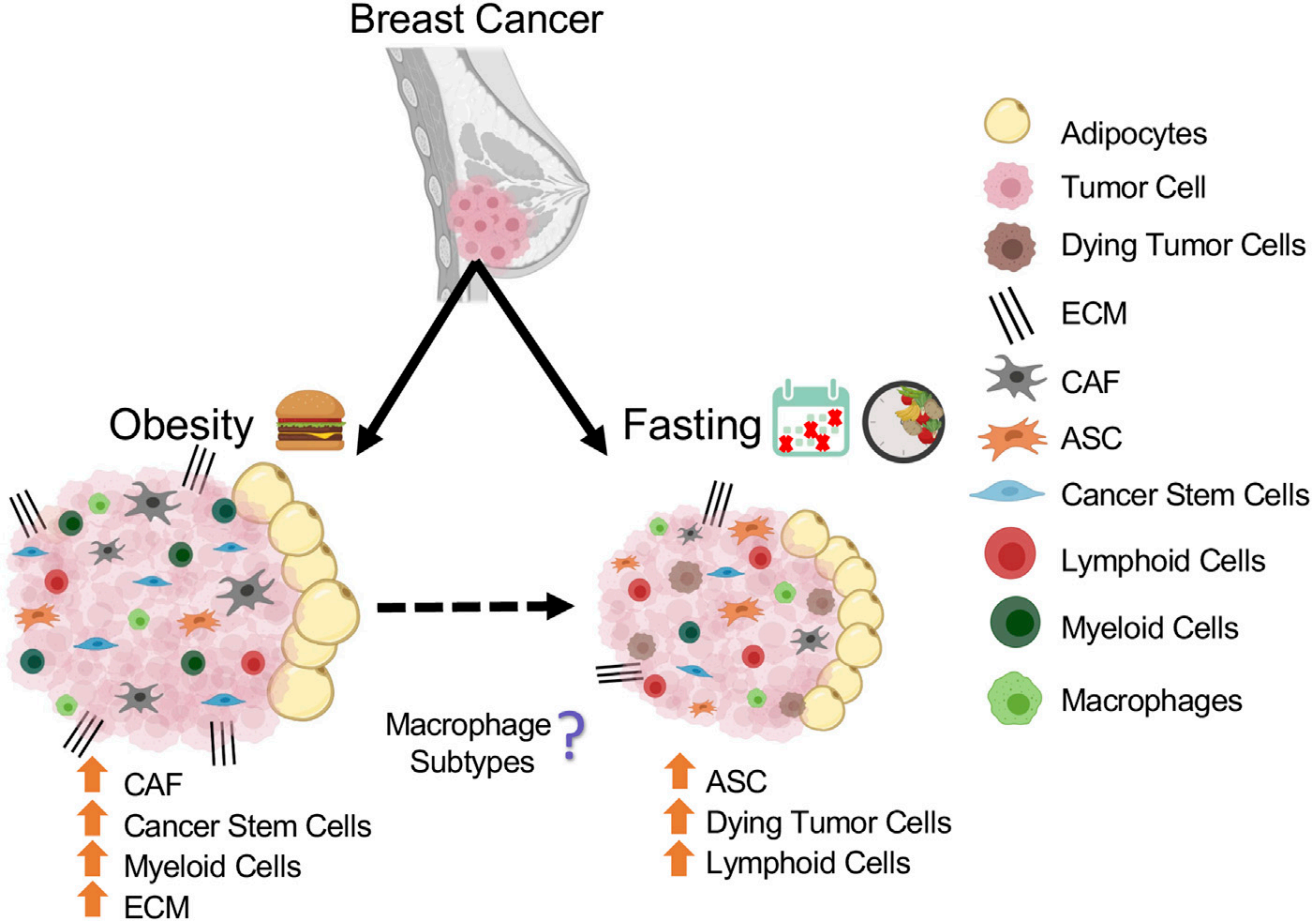
Schematic summarizing the population changes in mammary tumor microenvironment in response to obesity and fasting conditions.

In this review, we provided a detailed overview on of the effect of nutritional conditions such as obesity and fasting on ASCs, CAFs, immune cells, vascular cells, mammary epithelial, and cancer stem cells, all of which play an important role in the tumor microenvironment (Figure 2).

Studies have highlighted potential mechanisms responsible for modulating the tumor microenvironment in response to fasting. As immune surveillance is a critical component to killing cancer cells, most studies have examined the mechanism through which immune cell activity is modulated to enhance cytotoxicity. Ajona et al. demonstrated that the enhanced anti-tumor activity in response to fasting in combination with immune checkpoint blockade is attributed to decreased IGF-1 and IGF-1R in tumor cells. Subjecting mammary tumor-bearing mice to IGF-1R inhibitor and immune checkpoint blockade was sufficient to mimic the anti-tumor effect of fasting in lung cancer (Ajona et al., 2020). Furthermore, abolishing IGF-1 adequately mimicked prolonged fasting (48-h) and accelerated hematopoietic stem-cell protection and renewal following chemotherapy, thereby relieving immunosuppression (Cheng et al., 2014).

Although a reduction in IGF-1 is thought to be a leading candidate explaining fasting-induced tumor immunogenicity and improved therapeutic response, there are other possible mechanisms. Reduction in stress oxygenase enzyme Heme Oxygenase-1 by FMD increased immunogenicity via cytotoxic CD8+ T cells infiltration in mammary tumors and overall improved response to chemotherapy (Di Biase et al., 2016). On the other hand, FMD or fasting in HER2-negative breast cancer patients resulted in better mitigation of DNA damage in T-lymphocytes (De Groot et al., 2020). As immune cells are a key population responsible for anti-tumor activity, further research examining the mechanism behind fasting-induced immune cell changes are warranted.

Cancer cells and normal cells undergo differential stress resistance responses, and cancer cells are generally more sensitive to nutrient deprivation than normal cells (Raffaghello et al., 2008). Typically tumor cell survival is dependent on initiation of the “Warburg effect,” a phenomenon where the tumor cells experience high glucose uptake with lactate production under hypoxic conditions (Vander Heiden et al., 2009). This promotes ATP production through oxygen-independent pathways with reduced ROS-related DNA damage, contributing to chemoresistance (Zhou et al., 2010; Zhao et al., 2013). Bianchi et al. demonstrated that 48-h fasting promotes an anti-Warburg effect in colon cancer which drives glycolysis and oxidative phosphorylation to generate ROS and subsequent cancer cell apoptosis (Bianchi et al., 2015). This could explain the reported fasting-induced enhanced cancer cell sensitivity to chemotherapy and immunotherapy, which results in improved treatment response.

While the fasting effect has been investigated in the tumor immune cells, the impact fasting has on other cell populations in tumor is unknown. As discussed in this review, there are several potential mechanisms such as fasting-associated VEGF induction (Chen et al., 2017; Kim et al., 2017; Yang et al., 2018), AMPK activation (Kajita et al., 2008; Viscarra et al., 2011; Salminen et al., 2019), and downregulation of adipose-secreted leptin (Weigle et al., 1997; Pan et al., 2018) that can impact these individual populations. Since the tumor microenvironment is quite heterogeneous with various cell types, each population in the breast tissue plays an important role to contribute to disease pathogenesis. It is likely that a single mechanism is not responsible for modulating various cell populations in tumor microenvironment in response to fasting. Therefore, deepening our knowledge about how obesity and fasting can alter ASCs, CAFs, vascular cells, mammary epithelial, and cancer stem cells is essential to not only understand the microenvironmental transformation that undergoes with breast cancer, but also how to prevent it. Furthermore, expanding our understanding of possible mechanisms altering these populations could highlight novel targets that could be explored for cancer treatments.

Though not discussed in this review, a common effect that accompanies cancer progression is cancer cachexia. This occurs when cancer cells secrete factors that induce a hypermetabolic phenotype in adipose tissue and muscle tissue (Vazeille et al., 2017). As cancer patients commonly lose appetite, the hypermetabolic phenotype is often accompanied by inadequate caloric intake. This leads to drastic reductions in both lean and fat mass (Porporato, 2016). In fact, metastatic cancer patients with hypermetabolism associated with cancer cachexia exhibit reduced therapeutic response and survival (Vazeille et al., 2017; Baracos et al., 2018). As IF is known to increase resting energy expenditure and metabolic rate under non-tumorigenic conditions (Liu et al., 2019), it is possible that IF could have further adverse effects in metastatic cancer patients exhibiting cancer cachexia. Hence, studies in the future must closely examine the effect of IF on cancer cachexia.

Over recent years, new technologies have emerged to help provide a comprehensive detailed overview of tumor microenvironment interactions. Single-cell analyses such as high dimensional mass cytometry, known as CyTOF, utilize 40+ cell surface and intracellular markers to phenotypically and functionally characterize populations (Gadalla et al., 2019). Similarly, single-cell RNA or nuclei sequencing provides insight into the transcriptomic changes within each population in the microenvironment (Seow et al., 2020), also highlighting metabolic alterations (Xiao et al., 2019). Most recently, Jackson et al. revealed novel subgroups of breast cancer and associated cellular populations using single-cell analyses, highlighting the potential to use such techniques for targeted patient diagnosis and treatment (Jackson et al., 2020). These techniques have been used to investigate immune checkpoint blockade receptor expression on TIL (Beyrend et al., 2019), as well as to characterize and compare stromal cell heterogeneity between cancers (Qian et al., 2020). Future research utilizing these techniques will provide a unique opportunity to explore the tumor microenvironment in response to nutritional conditions such as obesity and fasting in an efficient and comprehensive manner (Jackson et al., 2020), with potential insight into each population.

Although many clinical studies have investigated the effect of fasting regimens in healthy, obese, and diabetic humans (Heilbronn et al., 2005; Harvie et al., 2011; Varady et al., 2013; Dorff et al., 2016; Gabel et al., 2018; Sutton et al., 2018; Hutchison et al., 2019; Lee et al., 2020; Wilkinson et al., 2020), there are few studies that have investigated the impact of fasting in cancer conditions. A 10-case series report examined the feasibility of fasting prior (48–140 h) and/or following chemotherapy (5–56 h) in cancer patients. Aside from hunger and light-headedness, no major side effects were reported. In fact, 6 patients reported a reduction in fatigue, weakness, and gastrointestinal side effects (Safdie et al., 2009). To further evaluate the quality of life in a randomized control study, Bauersfeld et al. conducted a trial where 34 ovarian and breast cancer patients were randomized to either a 60-h fasting duration (maximum 350 kcal), 36-h prior to chemotherapy and 24-h after chemotherapy, or control diet for the first 3 cycles of chemotherapy or second 3 cycles of chemotherapy. In addition to self-reported improved quality of life, decreased fatigue, and no major changes in weight, a greater benefit was observed with individuals subjected to fasting prior to chemotherapy in the first 3 cycles rather than later 3 cycles (Bauersfeld et al., 2018). Taken together, these studies suggest positive adherence and feasibility to fasting regimens in cancer patients.

In addition to the quality of life, a study of HER2-negative breast cancer patients with stage 2/3 breast cancer were randomized to 48-h fasting (24-h prior to and 24-h after chemotherapy) to evaluate chemotherapy-induced toxicity. Interestingly, erythrocyte and thrombocyte counts 1 week after chemotherapy were significantly greater in the fasted group, indicating reduced hematological toxicity and faster recovery of DNA damage (De Groot et al., 2015). Similarly, in an independent study, 48-h fasting prior to platinum-based chemotherapy or 72-h fasting (48-h prior to and 24-h after chemotherapy) resulted in decreased leukocyte DNA damage and a non-significant trend towards reduced neutropenia (Dorff et al., 2016). These studies are summarized in Table 1. Collectively, these studies suggest that fasting regimens in combination with current therapies are feasible and demonstrate positive benefits with mitigating toxicity.

**TABLE 1:** Summary of the outcomes and limitations of all clinical trials discussed in this review examining the effect of fasting on cancer.

Study	Sample population	Tumor model	Fasting schedule	Outcome	Limitations
Marinac et al. (2016)	- 2,413 breast cancer patients	- Breast cancer	- Time Restricted Feeding - nightly fasting (∼12.5 h/night)	- ↓ hemoglobin A_1c_ level	*- ERBB2* status was not available for a large portion of sample population
	- Age: 27–70 years			- ↑ nightly fasting <13 h was associated with reduced breast cancer recurrence	- Analyzed multiple primary endpoints for prognosis but did not control for multiple comparisons
	- No diabetes mellitus			- ↑ sleep duration	
De Groot et al. (2020)	- 129 patients with HER2-negative stage II/III breast cancer	- Breast Cancer	- FMD - 4-days of plant-based low amino-acid substitution diet followed by 3 days of ad libitum feeding	- ↓ DNA damage post chemotherapy	- ↓ compliance with each cycle of FMD
	- No diabetes mellitus			- ↑ sensitization to chemotherapy (increase in % tumor cell loss)	- Participants in the control group fasted some days impacting FMD analysis
				- ↑ response to radiological therapy	
De Groot et al. (2015)	- 13 patients with HER2-negative stage II/III breast cancer	- Breast Cancer	- Short-term fasting - 6 cycles of fasting 24 h prior and after start of chemotherapy (only allowed water, tea or coffee with no sugar)	- ↑ recovery of chemotherapy-induced DNA damage and toxicity in healthy cells	- Small sample size (2 participants withdrew after 3 cycles of FMD)
	- No diabetes mellitus			- Evidence of STF providing protection against chemotherapy-associated hematological toxicity	- High dose of dexamethasone was given during FMD cycles which can counteract the therapeutic effects of STF
	- Age ≥18 years			- ↓ in plasma IGF-1 which mediates protective effects for healthy cells	
	- Adequate bone marrow, renal, cardiac, and liver function				
Safdie et al. (2009)	- 10 patient case study (7 female, 3 male)	- Breast (4), Prostate (2), Ovarian (1), Non-small cell carcinoma of the lung (1), Esophageal adenocarcinoma (1)	- Short-term fasting - varying hours between 40–140 h in total prior chemotherapy and 5–56 h post chemotherapy compared to patients who did not fast	- ↓ reports of nausea, vomiting, diarrhea, abdominal cramps, and mucositis compared to control group fed *ad libitum*	- Inconsistent fasting periods
	- Median age: 61 years				- Medical reports between participants were reviewed retrospectively (eg. demographic information, diagnosis, treatment, imaging and laboratory analysis)
Bauersfeld et al. (2018)	- 34 patients	-Gynecological cancer (breast or ovarian cancer)	- Short-term fasting - 36 h prior to chemotherapy and 24 h post treatment (total 60 fasting hours)	- ↑ Quality of life (QoL) for fasted patients compared to control and reduce fatigue during chemotherapy	- Small sample size
	-Age ≥ 18 years				- Cross-over study design may produce carry-over effects and bias
	-No diabetes mellitus				- Study was conducted in Germany where there is a positive notion with the idea of fasting so participants may be biased to state that QoL has improved
	-Anticipated life expectancy >3 months				
Dorff et al. (2016)	- 20 patients	- Urothelial (bladder), ovarian or breast cancer	- Short-term fasting - 2 fasting cycles of each 24-h, 48-h and 72 h fasts consecutively compared to baseline measurements of participants	- ↑ hematopoietic protection with prolonged fasting periods	- Confounding variables as not all participants are diagnosed with similar stage of cancer progression
	- Median age: 61 years			- ↓ myelosuppression with fewer occurrences of neutropenia	- Incomplete compliance of fast since they were allowed “rescue food” of 200 kcal per day
	- 85% women				

STF, Short-Term Fasting; FMD, Fasting-Mimicking Diet.

Currently, there are many clinical trials undergoing to further examine the adherence, feasibility, and effectiveness of fasting in various cancers (Nencioni et al., 2018; U.S. National Library of Medicine, 2021). This research will shed light on whether fasting-associated benefits observed in preclinical models are translated into clinical settings, as well as the potential of fasting as a therapy. As the obesity epidemic increases worldwide, the global cancer burden is also expected to increase (Sung et al., 2021). Since fasting is a tangible, zero-cost, non-toxic, and easily applicable regimen, investigating the potential of fasting in the cancer setting is of significant value as current therapies are insufficient. Overall, this review provided a comprehensive overview of the current literature exploring obesity as well as fasting, with an emphasis on the mammary gland tissue and the development of breast cancer. As breast cancer is the most commonly diagnosed cancer (Sung et al., 2021), understanding how fasting regimens influence the mammary tumor microenvironment will provide insight into the mechanisms behind fasting-induced tumor benefits and can provide novel fasting-mimetics that can be easily translated in clinical settings.

## References

[B1] Ajona D. Ortiz-Espinosa S. Lozano T. Exposito F. Calvo A. Valencia K. (2020). Short-term Starvation Reduces IGF-1 Levels to Sensitize Lung Tumors to PD-1 Immune Checkpoint Blockade. Nat. Cancer 1, 75–85. 10.1038/s43018-019-0007-9 35121837

[B2] Al-Rawi N. Madkour M. Jahrami H. Salahat D. Alhasan F. Bahammam A. (2020). Effect of Diurnal Intermittent Fasting during Ramadan on Ghrelin, Leptin, Melatonin, and Cortisol Levels Among Overweight and Obese Subjects: A Prospective Observational Study. PLoS One 15, e0237922. 10.1371/journal.pone.0237922 32845924 PMC7449475

[B3] Almeneessier A. S. Pandi-Perumal S. R. Bahammam A. S. (2018). Intermittent Fasting, Insufficient Sleep, and Circadian Rhythm: Interaction and Effects on the Cardiometabolic System. Curr. Sleep Med. Rep 4, 179–195. 10.1007/s40675-018-0124-5

[B4] Baracos V. E. Martin L. Korc M. Guttridge D. C. Fearon K. C. H. (2018). Cancer-associated Cachexia. Nat. Rev. Dis. Primers 4, 17105. 10.1038/nrdp.2017.105 29345251

[B5] Bauersfeld S. P. Kessler C. S. Wischnewsky M. Jaensch A. Steckhan N. Stange R. (2018). The Effects of Short-Term Fasting on Quality of Life and Tolerance to Chemotherapy in Patients with Breast and Ovarian Cancer: a Randomized Cross-Over Pilot Study. BMC Cancer 18, 476. 10.1186/s12885-018-4353-2 29699509 PMC5921787

[B6] Beyrend G. Van Der Gracht E. Yilmaz A. Van Duikeren S. Camps M. Höllt T. (2019). PD-L1 Blockade Engages Tumor-Infiltrating Lymphocytes to Co-express Targetable Activating and Inhibitory Receptors. J. Immunotherapy Cancer 7, 217. 10.1186/s40425-019-0700-3 PMC6694641 31412943

[B7] Bianchi G. Martella R. Ravera S. Marini C. Capitanio S. Orengo A. (2015). Fasting Induces Anti-warburg Effect that Increases Respiration but Reduces ATP-Synthesis to Promote Apoptosis in colon Cancer Models. Oncotarget 6, 11806–11819. 10.18632/oncotarget.3688 25909219 PMC4494906

[B8] Bochet L. Lehuédé C. Dauvillier S. Wang Y. Y. Dirat B. Laurent V. (2013). Adipocyte-derived Fibroblasts Promote Tumor Progression and Contribute to the Desmoplastic Reaction in Breast Cancer. Cancer Res. 73, 5657–5668. 10.1158/0008-5472.CAN-13-0530 23903958

[B9] Buono R. Longo V. D. (2018). Starvation, Stress Resistance, and Cancer. Trends Endocrinol. Metab. 29, 271–280. 10.1016/j.tem.2018.01.008 29463451 PMC7477630

[B10] Buono R. Longo V. D. (2019). When Fasting Gets Tough, the Tough Immune Cells Get Going-Or Die. Cell 178, 1038–1040. 10.1016/j.cell.2019.07.052 31442398 PMC7474734

[B11] Carter J. M. Hoskin T. L. Pena M. A. Brahmbhatt R. Winham S. J. Frost M. H. (2018). Macrophagic "Crown-like Structures" Are Associated with an Increased Risk of Breast Cancer in Benign Breast Disease. Cancer Prev. Res. 11, 113–119. 10.1158/1940-6207.CAPR-17-0245 PMC7465518 29167285

[B12] Casteilla L. Planat-Benard V. Laharrague P. Cousin B. (2011). Adipose-derived Stromal Cells: Their Identity and Uses in Clinical Trials, an Update. Wjsc 3, 25–33. 10.4252/wjsc.v3.i4.25 21607134 PMC3097937

[B13] Castoldi A. Naffah De Souza C. Câmara N. O. S. Moraes-Vieira P. M. (2015). The Macrophage Switch in Obesity Development. Front. Immunol. 6, 637. 10.3389/fimmu.2015.00637 26779183 PMC4700258

[B14] Catterson J. H. Khericha M. Dyson M. C. Vincent A. J. Callard R. Haveron S. M. (2018). Short-Term, Intermittent Fasting Induces Long-Lasting Gut Health and TOR-independent Lifespan Extension. Curr. Biol. 28, 1714–1724. e1714. 10.1016/j.cub.2018.04.015 29779873 PMC5988561

[B15] Chamberlin T. D’Amato J. V. Arendt L. M. (2017). Obesity Reversibly Depletes the Basal Cell Population and Enhances Mammary Epithelial Cell Estrogen Receptor Alpha Expression and Progenitor Activity. Breast Cancer Res. 19, 128. 10.1186/s13058-017-0921-7 29187227 PMC5707907

[B16] ChÃ¡vez-GalÃ¡n L. Olleros M. L. Vesin D. Garcia I. (2015). Much More Than M1 and M2 Macrophages, There Are Also CD169+ and TCR+ Macrophages. Front. Immunol. 6, 263. 10.3389/fimmu.2015.00263 26074923 PMC4443739

[B17] Chen J. Sun X. Shao R. Xu Y. Gao J.-q. Liang W.-q. (2017). VEGF siRNA Delivered by Polycation Liposome-Encapsulated Calcium Phosphate Nanoparticles for Tumor Angiogenesis Inhibition in Breast Cancer. Ijn 12, 6075–6088. 10.2147/IJN.S142739 28860767 PMC5573052

[B18] Cheng C.-W. Adams G. B. Perin L. Wei M. Zhou X. Lam B. S. (2014). Prolonged Fasting Reduces IGF-1/PKA to Promote Hematopoietic-Stem-Cell-Based Regeneration and Reverse Immunosuppression. Cell Stem Cell 14, 810–823. 10.1016/j.stem.2014.04.014 24905167 PMC4102383

[B19] Cho Y. Hong N. Kim K.-w. Cho S. Lee M. Lee Y.-h. (2019). The Effectiveness of Intermittent Fasting to Reduce Body Mass Index and Glucose Metabolism: A Systematic Review and Meta-Analysis. Jcm 8, 1645. 10.3390/jcm8101645 PMC6832593 31601019

[B20] Chung S. J. Nagaraju G. P. Nagalingam A. Muniraj N. Kuppusamy P. Walker A. (2017). ADIPOQ/adiponectin Induces Cytotoxic Autophagy in Breast Cancer Cells through STK11/LKB1-Mediated Activation of the AMPK-ULK1 axis. Autophagy 13, 1386–1403. 10.1080/15548627.2017.1332565 28696138 PMC5584870

[B21] Clements V. K. Long T. Long R. Figley C. Smith D. M. C. Ostrand-Rosenberg S. (2018). Frontline Science: High Fat Diet and Leptin Promote Tumor Progression by Inducing Myeloid-Derived Suppressor Cells. J. Leukoc. Biol. 103, 395–407. 10.1002/JLB.4HI0517-210R 29345342 PMC7414791

[B22] Coats B. R. Schoenfelt K. Q. Barbosa-Lorenzi V. C. Peris E. Cui C. Hoffman A. (2017). Metabolically Activated Adipose Tissue Macrophages Perform Detrimental and Beneficial Functions during Diet-Induced Obesity. Cel Rep. 20, 3149–3161. 10.1016/j.celrep.2017.08.096 PMC5646237 28954231

[B23] Colleluori G. Perugini J. Barbatelli G. Cinti S. (2021). Mammary Gland Adipocytes in Lactation Cycle, Obesity and Breast Cancer. Rev. Endocr. Metab. Disord. 22, 241–255. 10.1007/s11154-021-09633-5 33751362 PMC8087566

[B24] Collins N. Han S.-J. Enamorado M. Link V. M. Huang B. Moseman E. A. (2019). The Bone Marrow Protects and Optimizes Immunological Memory during Dietary Restriction. Cell 178, 1088–1101. e1015. 10.1016/j.cell.2019.07.049 31442402 PMC6818271

[B25] Dang V. T. A. Tanabe K. Tanaka Y. Tokumoto N. Misumi T. Saeki Y. (2014). Fasting Enhances TRAIL-Mediated Liver Natural Killer Cell Activity via HSP70 Upregulation. PLoS One 9, e110748. 10.1371/journal.pone.0110748 25356750 PMC4214715

[B26] De Groot S. Lugtenberg R. T. Cohen D. Welters M. J. P. Ehsan I. Vreeswijk M. P. G. (2020). Fasting Mimicking Diet as an Adjunct to Neoadjuvant Chemotherapy for Breast Cancer in the Multicentre Randomized Phase 2 DIRECT Trial. Nat. Commun. 11, 3083. 10.1038/s41467-020-16138-3 32576828 PMC7311547

[B27] De Groot S. Vreeswijk M. P. Welters M. J. Gravesteijn G. Boei J. J. Jochems A. (2015). The Effects of Short-Term Fasting on Tolerance to (Neo) Adjuvant Chemotherapy in HER2-Negative Breast Cancer Patients: a Randomized Pilot Study. BMC Cancer 15, 652. 10.1186/s12885-015-1663-5 26438237 PMC4595051

[B28] De La Cruz Bonilla M. Stemler K. M. Jeter-Jones S. Fujimoto T. N. Molkentine J. Asencio Torres G. M. (2019). Fasting Reduces Intestinal Radiotoxicity, Enabling Dose-Escalated Radiation Therapy for Pancreatic Cancer. Int. J. Radiat. Oncology*Biology*Physics 105, 537–547. 10.1016/j.ijrobp.2019.06.2533 PMC6754784 31271824

[B29] De Lorenzo M. S. Baljinnyam E. Vatner D. E. Abarzua P. Vatner S. F. Rabson A. B. (2011). Caloric Restriction Reduces Growth of Mammary Tumors and Metastases. Carcinogenesis 32, 1381–1387. 10.1093/carcin/bgr107 21665891 PMC3165123

[B30] Divella R. De Luca R. Abbate I. Naglieri E. Daniele A. (2016). Obesity and Cancer: the Role of Adipose Tissue and Adipo-Cytokines-Induced Chronic Inflammation. J. Cancer 7, 2346–2359. 10.7150/jca.16884 27994674 PMC5166547

[B31] Di Biase S. Lee C. Brandhorst S. Manes B. Buono R. Cheng C.-W. (2016). Fasting-Mimicking Diet Reduces HO-1 to Promote T Cell-Mediated Tumor Cytotoxicity. Cancer Cell 30, 136–146. 10.1016/j.ccell.2016.06.005 27411588 PMC5388544

[B32] Dorff T. B. Groshen S. Garcia A. Shah M. Tsao-Wei D. Pham H. (2016). Safety and Feasibility of Fasting in Combination with Platinum-Based Chemotherapy. BMC Cancer 16, 360. 10.1186/s12885-016-2370-6 27282289 PMC4901417

[B33] Dunlap S. M. Chiao L. J. Nogueira L. Usary J. Perou C. M. Varticovski L. (2012). Dietary Energy Balance Modulates Epithelial-To-Mesenchymal Transition and Tumor Progression in Murine Claudin-Low and Basal-like Mammary Tumor Models. Cancer Prev. Res. 5, 930–942. 10.1158/1940-6207.CAPR-12-0034 PMC3822442 22588949

[B34] Ecker B. L. Lee J. Y. Sterner C. J. Solomon A. C. Pant D. K. Shen F. (2019). Impact of Obesity on Breast Cancer Recurrence and Minimal Residual Disease. Breast Cancer Res. 21, 41. 10.1186/s13058-018-1087-7 30867005 PMC6416940

[B35] Elias I. Franckhauser S. Ferré T. Vilà L. Tafuro S. Muñoz S. (2012). Adipose Tissue Overexpression of Vascular Endothelial Growth Factor Protects against Diet-Induced Obesity and Insulin Resistance. Diabetes 61, 1801–1813. 10.2337/db11-0832 22522611 PMC3379662

[B36] Fabbiano S. Suárez-Zamorano N. Rigo D. Veyrat-Durebex C. Stevanovic Dokic A. Colin D. J. (2016). Caloric Restriction Leads to Browning of White Adipose Tissue through Type 2 Immune Signaling. Cel Metab. 24, 434–446. 10.1016/j.cmet.2016.07.023 27568549

[B37] Faria S. S. Corrêa L. H. Heyn G. S. De Sant'ana L. P. Almeida R. d. N. Magalhães K. G. (2020). Obesity and Breast Cancer: The Role of Crown-Like Structures in Breast Adipose Tissue in Tumor Progression, Prognosis, and Therapy. J. Breast Cancer 23, 233–245. 10.4048/jbc.2020.23.e35 32595986 PMC7311368

[B38] Gabel K. Hoddy K. K. Haggerty N. Song J. Kroeger C. M. Trepanowski J. F. (2018). Effects of 8-hour Time Restricted Feeding on Body Weight and Metabolic Disease Risk Factors in Obese Adults: A Pilot Study. Nha 4, 345–353. 10.3233/NHA-170036 29951594 PMC6004924

[B39] Gadalla R. Noamani B. Macleod B. L. Dickson R. J. Guo M. Xu W. (2019). Validation of CyTOF against Flow Cytometry for Immunological Studies and Monitoring of Human Cancer Clinical Trials. Front. Oncol. 9, 415. 10.3389/fonc.2019.00415 31165047 PMC6534060

[B40] Goodman M. Liu Z. Zhu P. Li J. (2014). AMPK Activators as a Drug for Diabetes, Cancer and Cardiovascular Disease. Pharmaceut Reg. Aff. 03. 10.4172/2167-7689.1000118 PMC4966671 27478687

[B41] Hao J. Zhang Y. Yan X. Yan F. Sun Y. Zeng J. (2018). Circulating Adipose Fatty Acid Binding Protein Is a New Link Underlying Obesity-Associated Breast/Mammary Tumor Development. Cel Metab. 28, 689–705. 10.1016/j.cmet.2018.07.006 PMC6221972 30100196

[B42] Harvie M. N. Pegington M. Mattson M. P. Frystyk J. Dillon B. Evans G. (2011). The Effects of Intermittent or Continuous Energy Restriction on Weight Loss and Metabolic Disease Risk Markers: a Randomized Trial in Young Overweight Women. Int. J. Obes. 35, 714–727. 10.1038/ijo.2010.171 PMC3017674 20921964

[B43] Heilbronn L. K. Smith S. R. Martin C. K. Anton S. D. Ravussin E. (2005). Alternate-day Fasting in Nonobese Subjects: Effects on Body Weight, Body Composition, and Energy Metabolism. Am. J. Clin. Nutr. 81, 69–73. 10.1093/ajcn/81.1.69 15640462

[B44] Herold J. Kalucka J. (2020). Angiogenesis in Adipose Tissue: The Interplay between Adipose and Endothelial Cells. Front. Physiol. 11, 624903. 10.3389/fphys.2020.624903 33633579 PMC7900516

[B45] Hillers L. E. D'amato J. V. Chamberlin T. Paderta G. Arendt L. M. (2018). Obesity-Activated Adipose-Derived Stromal Cells Promote Breast Cancer Growth and Invasion. Neoplasia 20, 1161–1174. 10.1016/j.neo.2018.09.004 30317122 PMC6187054

[B46] Hillers-Ziemer L. E. Mcmahon R. Q. Hietpas M. Paderta G. Lebeau J. Mccready J. (2020). Obesity Promotes Cooperation of Cancer Stem-like Cells and Macrophages to Enhance Mammary Tumor Angiogenesis. Cancers 12, 502. 10.3390/cancers12020502 PMC7072330 32098183

[B47] Hsia L.-t. Ashley N. Ouaret D. Wang L. M. Wilding J. Bodmer W. F. (2016). Myofibroblasts Are Distinguished from Activated Skin Fibroblasts by the Expression of AOC3 and Other Associated Markers. Proc. Natl. Acad. Sci. USA 113, E2162–E2171. 10.1073/pnas.1603534113 27036009 PMC4839407

[B48] Hua L. Li J. Feng B. Jiang D. Jiang X. Luo T. (2021). Dietary Intake Regulates White Adipose Tissues Angiogenesis via Liver Fibroblast Growth Factor 21 in Male Mice. Endocrinology 162, bqaa244. 10.1210/endocr/bqaa244 33369618 PMC7814301

[B49] Hutchison A. T. Regmi P. Manoogian E. N. C. Fleischer J. G. Wittert G. A. Panda S. (2019). Time‐Restricted Feeding Improves Glucose Tolerance in Men at Risk for Type 2 Diabetes: A Randomized Crossover Trial. Obesity 27, 724–732. 10.1002/oby.22449 31002478

[B50] Incio J. Ligibel J. A. Mcmanus D. T. Suboj P. Jung K. Kawaguchi K. (2018). Obesity Promotes Resistance to Anti-VEGF Therapy in Breast Cancer by Up-Regulating IL-6 and Potentially FGF-2. Sci. Transl. Med. 10, eaag0945. 10.1126/scitranslmed.aag0945 29540614 PMC5936748

[B51] Inoue S.-i. Takahashi K. Okumura-Noda H. Kinoshita T. (2016). Auxin Influx Carrier AUX1 Confers Acid Resistance for Arabidopsis Root Elongation through the Regulation of Plasma Membrane H+-ATPase. Plant Cel Physiol 57, 2194–2201. 10.1093/pcp/pcw136 PMC5434668 27503216

[B52] Jackson H. W. Fischer J. R. Zanotelli V. R. T. Ali H. R. Mechera R. Soysal S. D. (2020). The Single-Cell Pathology Landscape of Breast Cancer. Nature 578, 615–620. 10.1038/s41586-019-1876-x 31959985

[B53] Jeong H. Hwang I. Kang S. H. Shin H. C. Kwon S. Y. (2019). Tumor-Associated Macrophages as Potential Prognostic Biomarkers of Invasive Breast Cancer. J. Breast Cancer 22, 38–51. 10.4048/jbc.2019.22.e5 30941232 PMC6438840

[B54] Ji Y. Sun S. Xia S. Yang L. Li X. Qi L. (2012). Short Term High Fat Diet challenge Promotes Alternative Macrophage Polarization in Adipose Tissue via Natural Killer T Cells and Interleukin-4. J. Biol. Chem. 287, 24378–24386. 10.1074/jbc.M112.371807 22645141 PMC3397864

[B55] Kado T. Nawaz A. Takikawa A. Usui I. Tobe K. (2019). Linkage of CD8+ T Cell Exhaustion with High-Fat Diet-Induced Tumourigenesis. Sci. Rep. 9, 12284. 10.1038/s41598-019-48678-0 31439906 PMC6706391

[B56] Kahounová Z. Kurfürstová D. Bouchal J. Kharaishvili G. Navrátil J. Remšík J. (2018). The Fibroblast Surface Markers FAP, Anti-fibroblast, and FSP Are Expressed by Cells of Epithelial Origin and May Be Altered during Epithelial-To-Mesenchymal Transition. Cytometry 93, 941–951. 10.1002/cyto.a.23101 28383825

[B57] Kajita K. Mune T. Ikeda T. Matsumoto M. Uno Y. Sugiyama C. (2008). Effect of Fasting on PPARγ and AMPK Activity in Adipocytes. Diabetes Res. Clin. Pract. 81, 144–149. 10.1016/j.diabres.2008.05.003 18562038

[B58] Karagiannides I. Thomou T. Tchkonia T. Pirtskhalava T. Kypreos K. E. Cartwright A. (2006). Increased CUG Triplet Repeat-Binding Protein-1 Predisposes to Impaired Adipogenesis with Aging. J. Biol. Chem. 281, 23025–23033. 10.1074/jbc.M513187200 16754681

[B59] Kim K.-H. Kim Y. H. Son J. E. Lee J. H. Kim S. Choe M. S. (2017). Intermittent Fasting Promotes Adipose Thermogenesis and Metabolic Homeostasis via VEGF-Mediated Alternative Activation of Macrophage. Cell Res 27, 1309–1326. 10.1038/cr.2017.126 29039412 PMC5674160

[B60] Kim Y. H. Lee J. H. Yeung J. L.-H. Das E. Kim R. Y. Jiang Y. (2019). Thermogenesis-independent Metabolic Benefits Conferred by Isocaloric Intermittent Fasting in Ob/ob Mice. Sci. Rep. 9, 2479. 10.1038/s41598-019-39380-2 30792482 PMC6385507

[B61] Klempel M. C. Kroeger C. M. Varady K. A. (2013). Alternate Day Fasting (ADF) with a High-Fat Diet Produces Similar Weight Loss and Cardio-protection as ADF with a Low-Fat Diet. Metabolism 62, 137–143. 10.1016/j.metabol.2012.07.002 22889512

[B62] Kolb R. Kluz P. Tan Z. W. Borcherding N. Bormann N. Vishwakarma A. (2019). Obesity-associated Inflammation Promotes Angiogenesis and Breast Cancer via Angiopoietin-like 4. Oncogene 38, 2351–2363. 10.1038/s41388-018-0592-6 30518876 PMC6440811

[B63] Kolb R. Zhang W. (2020). Obesity and Breast Cancer: A Case of Inflamed Adipose Tissue. Cancers 12, 1686. 10.3390/cancers12061686 PMC7352736 32630445

[B64] Lechner S. Mitterberger M. C. Mattesich M. Zwerschke W. (2013). Role of C/EBPβ-LAP and C/EBPβ-LIP in Early Adipogenic Differentiation of Human white Adipose-Derived Progenitors and at Later Stages in Immature Adipocytes. Differentiation 85, 20–31. 10.1016/j.diff.2012.11.001 23314288

[B65] Lee B.-C. Kim M.-S. Pae M. Yamamoto Y. Eberlé D. Shimada T. (2016). Adipose Natural Killer Cells Regulate Adipose Tissue Macrophages to Promote Insulin Resistance in Obesity. Cel Metab. 23, 685–698. 10.1016/j.cmet.2016.03.002 PMC4833527 27050305

[B66] Lee C. Raffaghello L. Brandhorst S. Safdie F. M. Bianchi G. Martin-Montalvo A. (2012). Fasting Cycles Retard Growth of Tumors and Sensitize a Range of Cancer Cell Types to Chemotherapy. Sci. Transl. Med. 4, 124ra127. 10.1126/scitranslmed.3003293 PMC3608686 22323820

[B67] Lee J. H. Verma N. Thakkar N. Yeung C. Sung H.-K. (2020). Intermittent Fasting: Physiological Implications on Outcomes in Mice and Men. Physiology 35, 185–195. 10.1152/physiol.00030.2019 32293230

[B68] Lee K.-H. Hsu E.-C. Guh J.-H. Yang H.-C. Wang D. Kulp S. K. (2011). Targeting Energy Metabolic and Oncogenic Signaling Pathways in Triple-Negative Breast Cancer by a Novel Adenosine Monophosphate-Activated Protein Kinase (AMPK) Activator. J. Biol. Chem. 286, 39247–39258. 10.1074/jbc.M111.264598 21917926 PMC3234749

[B69] Li G. Xie C. Lu S. Nichols R. G. Tian Y. Li L. (2017). Intermittent Fasting Promotes White Adipose Browning and Decreases Obesity by Shaping the Gut Microbiota. Cel Metab. 26, 672–685. 10.1016/j.cmet.2017.08.019 PMC5668683 28918936

[B70] Lin Y. Li Q. (2007). Expression and Function of Leptin and its Receptor in Mouse Mammary Gland. Sci. China Ser. C 50, 669–675. 10.1007/s11427-007-0077-2 17879067

[B71] Liu B. Page A. J. Hutchison A. T. Wittert G. A. Heilbronn L. K. (2019). Intermittent Fasting Increases Energy Expenditure and Promotes Adipose Tissue browning in Mice. Nutrition 66, 38–43. 10.1016/j.nut.2019.03.015 31207437

[B72] Lumeng C. N. Bodzin J. L. Saltiel A. R. (2007). Obesity Induces a Phenotypic Switch in Adipose Tissue Macrophage Polarization. J. Clin. Invest. 117, 175–184. 10.1172/JCI29881 17200717 PMC1716210

[B73] Mao T. Wei Q. Zhao F. Zhang C. (2021). Short-term Fasting Reshapes Fat Tissue. Endocr. J. 68, 387–398. 10.1507/endocrj.EJ20-0405 33441502

[B74] Marinac C. R. Natarajan L. Sears D. D. Gallo L. C. Hartman S. J. Arredondo E. (2015a). Prolonged Nightly Fasting and Breast Cancer Risk: Findings from NHANES (2009-2010). Cancer Epidemiol. Biomarkers Prev. 24, 783–789. 10.1158/1055-9965.EPI-14-1292 25896523 PMC4417458

[B75] Marinac C. R. Nelson S. H. Breen C. I. Hartman S. J. Natarajan L. Pierce J. P. (2016). Prolonged Nightly Fasting and Breast Cancer Prognosis. JAMA Oncol. 2, 1049–1055. 10.1001/jamaoncol.2016.0164 27032109 PMC4982776

[B76] Marinac C. R. Sears D. D. Natarajan L. Gallo L. C. Breen C. I. Patterson R. E. (2015b). Frequency and Circadian Timing of Eating May Influence Biomarkers of Inflammation and Insulin Resistance Associated with Breast Cancer Risk. PLoS One 10, e0136240. 10.1371/journal.pone.0136240 26305095 PMC4549297

[B77] Martens A. Wistuba-Hamprecht K. Foppen M. G. Yuan J. Postow M. A. Wong P. (2016). Baseline Peripheral Blood Biomarkers Associated with Clinical Outcome of Advanced Melanoma Patients Treated with Ipilimumab. Clin. Cancer Res. 22, 2908–2918. 10.1158/1078-0432.CCR-15-2412 26787752 PMC5770142

[B78] Mishra A. K. Parish C. R. Wong M.-L. Licinio J. Blackburn A. C. (2017). Leptin Signals via TGFB1 to Promote Metastatic Potential and Stemness in Breast Cancer. PLoS One 12, e0178454. 10.1371/journal.pone.0178454 28542577 PMC5444832

[B79] Moro T. Tinsley G. Bianco A. Marcolin G. Pacelli Q. F. Battaglia G. (2016). Effects of Eight Weeks of Time-Restricted Feeding (16/8) on Basal Metabolism, Maximal Strength, Body Composition, Inflammation, and Cardiovascular Risk Factors in Resistance-Trained Males. J. Transl Med. 14, 290. 10.1186/s12967-016-1044-0 27737674 PMC5064803

[B80] Munsell M. F. Sprague B. L. Berry D. A. Chisholm G. Trentham-Dietz A. (2014). Body Mass index and Breast Cancer Risk According to Postmenopausal Estrogen-Progestin Use and Hormone Receptor Status. Epidemiol. Rev. 36, 114–136. 10.1093/epirev/mxt010 24375928 PMC3873844

[B81] Mustafi D. Fernandez S. Markiewicz E. Fan X. Zamora M. Mueller J. (2017). MRI Reveals Increased Tumorigenesis Following High Fat Feeding in a Mouse Model of Triple-Negative Breast Cancer. NMR Biomed. 30, e3758. 10.1002/nbm.3758 PMC5764539 28661075

[B82] Nagai M. Noguchi R. Takahashi D. Morikawa T. Koshida K. Komiyama S. (2019). Fasting-Refeeding Impacts Immune Cell Dynamics and Mucosal Immune Responses. Cell 178, 1072–1087. e1014. 10.1016/j.cell.2019.07.047 31442401

[B83] Narita T. Kobayashi M. Itakura K. Itagawa R. Kabaya R. Sudo Y. (2018). Differential Response to Caloric Restriction of Retroperitoneal, Epididymal, and Subcutaneous Adipose Tissue Depots in Rats. Exp. Gerontol. 104, 127–137. 10.1016/j.exger.2018.01.016 29410017

[B84] Nencioni A. Caffa I. Cortellino S. Longo V. D. (2018). Fasting and Cancer: Molecular Mechanisms and Clinical Application. Nat. Rev. Cancer 18, 707–719. 10.1038/s41568-018-0061-0 30327499 PMC6938162

[B85] Novak M. L. Koh T. J. (2013). Macrophage Phenotypes during Tissue Repair. J. Leukoc. Biol. 93, 875–881. 10.1189/jlb.1012512 23505314 PMC3656331

[B86] Ohmura K. Ishimori N. Ohmura Y. Tokuhara S. Nozawa A. Horii S. (2010). Natural Killer T Cells Are Involved in Adipose Tissues Inflammation and Glucose Intolerance in Diet-Induced Obese Mice. Atvb 30, 193–199. 10.1161/ATVBAHA.109.198614 19910631

[B87] Olea-Flores M. Zuñiga-Eulogio M. Tacuba-Saavedra A. Bueno-Salgado M. Sánchez-Carvajal A. Vargas-Santiago Y. (2019). Leptin Promotes Expression of EMT-Related Transcription Factors and Invasion in a Src and FAK-dependent Pathway in MCF10A Mammary Epithelial Cells. Cells 8, 1133. 10.3390/cells8101133 PMC6829404 31554180

[B88] Olson L. K. Tan Y. Zhao Y. Aupperlee M. D. Haslam S. Z. (2010). Pubertal Exposure to High Fat Diet Causes Mouse Strain-dependent Alterations in Mammary Gland Development and Estrogen Responsiveness. Int. J. Obes. 34, 1415–1426. 10.1038/ijo.2010.51 PMC2923244 20231845

[B89] Onesti C. E. Josse C. Boulet D. Thiry J. Beaumecker B. Bours V. (2020). The Relative Eosinophil Count in Breast Cancer as an Emerging Prognostic Biomarker. Eur. J. Cancer 138, 1761176. 10.1016/S0959-8049(20)30766-8 PMC7458605 32923121

[B90] Pajares B. Pollán M. Martín M. Mackey J. R. Lluch A. Gavila J. (2013). Obesity and Survival in Operable Breast Cancer Patients Treated with Adjuvant Anthracyclines and Taxanes According to Pathological Subtypes: a Pooled Analysis. Breast Cancer Res. 15, R105. 10.1186/bcr3572 24192331 PMC3978725

[B91] Pan H. Deng L.-L. Cui J.-Q. Shi L. Yang Y.-C. Luo J.-H. (2018). Association between Serum Leptin Levels and Breast Cancer Risk. Medicine (Baltimore) 97, e11345. 10.1097/MD.0000000000011345 29979411 PMC6076146

[B92] Park C. C. Bissell M. J. Barcellos-Hoff M. H. (2000). The Influence of the Microenvironment on the Malignant Phenotype. Mol. Med. Today 6, 324–329. 10.1016/s1357-4310(00)01756-1 10904250

[B93] Park J. Kim M. Sun K. An Y. A. Gu X. Scherer P. E. (2017). VEGF-A-Expressing Adipose Tissue Shows Rapid Beiging and Enhanced Survival after Transplantation and Confers IL-4-Independent Metabolic Improvements. Diabetes 66, 1479–1490. 10.2337/db16-1081 28254844 PMC5440018

[B94] Porporato P. E. (2016). Understanding Cachexia as a Cancer Metabolism Syndrome. Oncogenesis 5, e200. 10.1038/oncsis.2016.3 26900952 PMC5154342

[B95] Qian J. Olbrecht S. Boeckx B. Vos H. Laoui D. Etlioglu E. (2020). A Pan-Cancer Blueprint of the Heterogeneous Tumor Microenvironment Revealed by Single-Cell Profiling. Cel Res 30, 745–762. 10.1038/s41422-020-0355-0 PMC7608385 32561858

[B96] Quail D. F. Dannenberg A. J. (2019). The Obese Adipose Tissue Microenvironment in Cancer Development and Progression. Nat. Rev. Endocrinol. 15, 139–154. 10.1038/s41574-018-0126-x 30459447 PMC6374176

[B97] Raffaghello L. Lee C. Safdie F. M. Wei M. Madia F. Bianchi G. (2008). Starvation-dependent Differential Stress Resistance Protects normal but Not Cancer Cells against High-Dose Chemotherapy. Proc. Natl. Acad. Sci. 105, 8215–8220. 10.1073/pnas.0708100105 18378900 PMC2448817

[B98] Ramamonjisoa N. Ackerstaff E. (2017). Characterization of the Tumor Microenvironment and Tumor-Stroma Interaction by Non-invasive Preclinical Imaging. Front. Oncol. 7, 3. 10.3389/fonc.2017.00003 28197395 PMC5281579

[B99] Rangan P. Choi I. Wei M. Navarrete G. Guen E. Brandhorst S. (2019). Fasting-Mimicking Diet Modulates Microbiota and Promotes Intestinal Regeneration to Reduce Inflammatory Bowel Disease Pathology. Cel Rep. 26, 2704–2719. e2706. 10.1016/j.celrep.2019.02.019 PMC6528490 30840892

[B100] Raut P. K. Kim S.-H. Choi D. Y. Jeong G.-S. Park P.-H. (2019). Growth of Breast Cancer Cells by Leptin Is Mediated via Activation of the Inflammasome: Critical Roles of Estrogen Receptor Signaling and Reactive Oxygen Species Production. Biochem. Pharmacol. 161, 73–88. 10.1016/j.bcp.2019.01.006 30633869

[B101] Revelo X. S. Luck H. Winer S. Winer D. A. (2014). Morphological and Inflammatory Changes in Visceral Adipose Tissue during Obesity. Endocr. Pathol. 25, 93–101. 10.1007/s12022-013-9288-1 24356782

[B102] Ringel A. E. Drijvers J. M. Baker G. J. Catozzi A. García-Cañaveras J. C. Gassaway B. M. (2020). Obesity Shapes Metabolism in the Tumor Microenvironment to Suppress Anti-tumor Immunity. Cell 183, 1848–1866. 10.1016/j.cell.2020.11.009 33301708 PMC8064125

[B103] Ruiz-Ojeda F. J. Méndez-Gutiérrez A. Aguilera C. M. Plaza-Díaz J. (2019). Extracellular Matrix Remodeling of Adipose Tissue in Obesity and Metabolic Diseases. Ijms 20, 4888. 10.3390/ijms20194888 PMC6801592 31581657

[B104] Ruppert P. M. M. Michielsen C. C. J. R. Hazebroek E. J. Pirayesh A. Olivecrona G. Afman L. A. (2020). Fasting Induces ANGPTL4 and Reduces LPL Activity in Human Adipose Tissue. Mol. Metab. 40, 101033. 10.1016/j.molmet.2020.101033 32504883 PMC7334813

[B105] Safdie F. M. Dorff T. Quinn D. Fontana L. Wei M. Lee C. (2009). Fasting and Cancer Treatment in Humans: A Case Series Report. Aging 1, 988–1007. 10.18632/aging.100114 20157582 PMC2815756

[B106] Sahai E. Astsaturov I. Cukierman E. Denardo D. G. Egeblad M. Evans R. M. (2020). A Framework for Advancing Our Understanding of Cancer-Associated Fibroblasts. Nat. Rev. Cancer 20, 174–186. 10.1038/s41568-019-0238-1 31980749 PMC7046529

[B107] Salminen A. Kauppinen A. Kaarniranta K. (2019). AMPK Activation Inhibits the Functions of Myeloid-Derived Suppressor Cells (MDSC): Impact on Cancer and Aging. J. Mol. Med. 97, 1049–1064. 10.1007/s00109-019-01795-9 31129755 PMC6647228

[B108] Seow J. J. W. Wong R. M. M. Pai R. Sharma A. (2020). Single‐Cell RNA Sequencing for Precision Oncology: Current State-Of-Art. J. Indian Inst. Sci. 100, 579–588. 10.1007/s41745-020-00178-1 32837038 PMC7264973

[B109] Shao M. Vishvanath L. Busbuso N. C. Hepler C. Shan B. Sharma A. X. (2018). De Novo adipocyte Differentiation from Pdgfrβ+ Preadipocytes Protects against Pathologic Visceral Adipose Expansion in Obesity. Nat. Commun. 9, 890. 10.1038/s41467-018-03196-x 29497032 PMC5832777

[B110] Shushimita S. De Bruijn M. J. W. De Bruin R. W. F. IJzermans J. N. M. Hendriks R. W. Dor F. J. M. F. (2014). Dietary Restriction and Fasting Arrest B and T Cell Development and Increase Mature B and T Cell Numbers in Bone Marrow. PLoS One 9, e87772. 10.1371/journal.pone.0087772 24504160 PMC3913690

[B111] Simon S. C. S. Hu X. Panten J. Grees M. Renders S. Thomas D. (2020). Eosinophil Accumulation Predicts Response to Melanoma Treatment with Immune Checkpoint Inhibitors. Oncoimmunology 9, 1727116. 10.1080/2162402X.2020.1727116 32117594 PMC7028332

[B112] Singh R. Parveen M. Basgen J. M. Fazel S. Meshesha M. F. Thames E. C. (2016). Increased Expression of Beige/Brown Adipose Markers from Host and Breast Cancer Cells Influence Xenograft Formation in Mice. Mol. Cancer Res. 14, 78–92. 10.1158/1541-7786.MCR-15-0151 26464213 PMC4749269

[B113] Speaker K. J. Paton M. M. Cox S. S. Fleshner M. (2016). A Single Bout of Fasting (24 H) Reduces Basal Cytokine Expression and Minimally Impacts the Sterile Inflammatory Response in the White Adipose Tissue of Normal Weight F344 Rats. Mediators Inflamm. 2016, 1–13. 10.1155/2016/1698071 PMC5203907 28077915

[B114] Spielmann J. Mattheis L. Jung J.-S. Rausse H. Glass M. Bähr I. (2020). Effects of Obesity on NK Cells in a Mouse Model of Postmenopausal Breast Cancer. Sci. Rep. 10, 20606. 10.1038/s41598-020-76906-5 33244094 PMC7692502

[B115] Strissel K. J. Defuria J. Shaul M. E. Bennett G. Greenberg A. S. Obin M. S. (2010). T-cell Recruitment and Th1 Polarization in Adipose Tissue during Diet-Induced Obesity in C57BL/6 Mice. Obesity (Silver Spring) 18, 1918–1925. 10.1038/oby.2010.1 20111012 PMC2894258

[B116] Strong A. L. Pei D. T. Hurst C. G. Gimble J. M. Burow M. E. Bunnell B. A. (2017). Obesity Enhances the Conversion of Adipose-Derived Stromal/Stem Cells into Carcinoma-Associated Fibroblast Leading to Cancer Cell Proliferation and Progression to an Invasive Phenotype. Stem Cell Int. 2017, 1–11. 10.1155/2017/9216502 PMC5748106 29527228

[B117] Sun K. Asterholm I. W. Kusminski C. M. Bueno A. C. Wang Z. V. Pollard J. W. (2012). Dichotomous Effects of VEGF-A on Adipose Tissue Dysfunction. Proc. Natl. Acad. Sci. 109, 5874–5879. 10.1073/pnas.1200447109 22451920 PMC3326476

[B118] Sundaram S. Yan L. (2018). Time-restricted Feeding Mitigates High-Fat Diet-Enhanced Mammary Tumorigenesis in MMTV-PyMT Mice. Nutr. Res. 59, 72–79. 10.1016/j.nutres.2018.07.014 30442235

[B119] Sung H.-K. Doh K.-O. Son J. E. Park J. G. Bae Y. Choi S. (2013). Adipose Vascular Endothelial Growth Factor Regulates Metabolic Homeostasis through Angiogenesis. Cel Metab. 17, 61–72. 10.1016/j.cmet.2012.12.010 23312284

[B120] Sung H. Ferlay J. Siegel R. L. Laversanne M. Soerjomataram I. Jemal A. (2021). Global Cancer Statistics 2020: GLOBOCAN Estimates of Incidence and Mortality Worldwide for 36 Cancers in 185 Countries. CA A. Cancer J. Clin. 71, 209–249. 10.3322/caac.21660 33538338

[B121] Sutton E. F. Beyl R. Early K. S. Cefalu W. T. Ravussin E. Peterson C. M. (2018). Early Time-Restricted Feeding Improves Insulin Sensitivity, Blood Pressure, and Oxidative Stress Even without Weight Loss in Men with Prediabetes. Cel Metab. 27, 1212–1221. e1213. 10.1016/j.cmet.2018.04.010 PMC5990470 29754952

[B122] Tang H.-N. Tang C.-Y. Man X.-F. Tan S.-W. Guo Y. Tang J. (2017). Plasticity of Adipose Tissue in Response to Fasting and Refeeding in Male Mice. Nutr. Metab. (Lond) 14, 3. 10.1186/s12986-016-0159-x 28070205 PMC5217231

[B123] Tchkonia T. Pirtskhalava T. Thomou T. Cartwright M. J. Wise B. Karagiannides I. (2007). Increased TNFα and CCAAT/enhancer-binding Protein Homologous Protein with Aging Predispose Preadipocytes to Resist Adipogenesis. Am. J. Physiology-Endocrinology Metab. 293, E1810–E1819. 10.1152/ajpendo.00295.2007 17911345

[B124] Tenvooren I. Jenks M. Z. Rashid H. Cook K. L. Muhlemann J. K. Sistrunk C. (2019). Elevated Leptin Disrupts Epithelial Polarity and Promotes Premalignant Alterations in the Mammary Gland. Oncogene 38, 3855–3870. 10.1038/s41388-019-0687-8 30670780 PMC6525037

[B125] Trepanowski J. F. Kroeger C. M. Barnosky A. Klempel M. Bhutani S. Hoddy K. K. (2018). Effects of Alternate-Day Fasting or Daily Calorie Restriction on Body Composition, Fat Distribution, and Circulating Adipokines: Secondary Analysis of a Randomized Controlled Trial. Clin. Nutr. 37, 1871–1878. 10.1016/j.clnu.2017.11.018 29258678 PMC5988907

[B126] Turbitt W. J. Collins S. D. Meng H. Rogers C. J. (2019). Increased Adiposity Enhances the Accumulation of MDSCs in the Tumor Microenvironment and Adipose Tissue of Pancreatic Tumor-Bearing Mice and in Immune Organs of Tumor-free Hosts. Nutrients 11, 3012. 10.3390/nu11123012 PMC6950402 31835454

[B127] Tzanavari T. Giannogonas P. Karalis K. P. (2010). TNF-α and Obesity. Curr. Dir. Autoimmun. 11, 145–156. 10.1159/000289203 20173393

[B128] Ullah R. Su Y. Shen Y. Li C. Xu X. Zhang J. (2017). Postnatal Feeding with High-Fat Diet Induces Obesity and Precocious Puberty in C57BL/6J Mouse Pups: a Novel Model of Obesity and Puberty. Front. Med. 11, 266–276. 10.1007/s11684-017-0530-y 28500430

[B129] U.S. National Library of Medicine (2021). Search of: Fasting, Cancer - List Results - ClinicalTrials.Gov [Online]. ClinicalTrials.gov: NIH: U.S. National Library of Medicine. Available: https://clinicaltrials.gov/ct2/results?cond=fasting+and+cancer&term=&cntry=&state=&city=&dist= (Accessed August 20, 2021).

[B130] Vander Heiden M. G. Cantley L. C. Thompson C. B. (2009). Understanding the Warburg Effect: the Metabolic Requirements of Cell Proliferation. Science 324, 1029–1033. 10.1126/science.1160809 19460998 PMC2849637

[B131] Varady K. A. Allister C. A. Roohk D. J. Hellerstein M. K. (2010). Improvements in Body Fat Distribution and Circulating Adiponectin by Alternate-Day Fasting versus Calorie Restriction☆. J. Nutr. Biochem. 21, 188–195. 10.1016/j.jnutbio.2008.11.001 19195863

[B132] Varady K. A. Bhutani S. Klempel M. C. Kroeger C. M. Trepanowski J. F. Haus J. M. (2013). Alternate Day Fasting for Weight Loss in normal Weight and Overweight Subjects: a Randomized Controlled Trial. Nutr. J. 12, 146. 10.1186/1475-2891-12-146 24215592 PMC3833266

[B133] Varady K. A. Roohk D. J. McEvoy‐Hein B. K. Gaylinn B. D. Thorner M. O. Hellerstein M. K. (2008). Modified Alternate‐day Fasting Regimens Reduce Cell Proliferation Rates to a Similar Extent as Daily Calorie Restriction in Mice. FASEB j. 22, 2090–2096. 10.1096/fj.07-098178 18184721 PMC2975447

[B134] Vazeille C. Jouinot A. Durand J.-P. Neveux N. Boudou-Rouquette P. Huillard O. (2017). Relation between Hypermetabolism, Cachexia, and Survival in Cancer Patients: a Prospective Study in 390 Cancer Patients before Initiation of Anticancer Therapy. Am. J. Clin. Nutr. 105, 1139–1147. 10.3945/ajcn.116.140434 28356274

[B135] Verma N. Thakkar N. Phillips J. Ealey K. Sung H.-K. (2020). Dynamic Remodeling of white Adipose Tissue by Intermittent Fasting. Curr. Opin. Food Sci. 34, 21–29. 10.1016/j.cofs.2020.10.023

[B136] Vila I. K. Badin P.-M. Marques M.-A. Monbrun L. Lefort C. Mir L. (2014). Immune Cell Toll-like Receptor 4 Mediates the Development of Obesity- and Endotoxemia-Associated Adipose Tissue Fibrosis. Cel Rep. 7, 1116–1129. 10.1016/j.celrep.2014.03.062 24794440

[B137] Viscarra J. A. Champagne C. D. Crocker D. E. Ortiz R. M. (2011). 5′AMP-activated Protein Kinase Activity Is Increased in Adipose Tissue of Northern Elephant Seal Pups during Prolonged Fasting-Induced Insulin Resistance. J. Endocrinol. 209, 317–325. 10.1530/JOE-11-0017 21429964 PMC3250370

[B138] Wang L. Cao L. Wang H. Liu B. Zhang Q. Meng Z. (2017). Cancer-associated Fibroblasts Enhance Metastatic Potential of Lung Cancer Cells through IL-6/STAT3 Signaling Pathway. Oncotarget 8, 76116–76128. 10.18632/oncotarget.18814 29100297 PMC5652691

[B139] Wei J. Ghosh A. K. Sargent J. L. Komura K. Wu M. Huang Q.-Q. (2010). PPARγ Downregulation by TGFß in Fibroblast and Impaired Expression and Function in Systemic Sclerosis: A Novel Mechanism for Progressive Fibrogenesis. PLoS One 5, e13778. 10.1371/journal.pone.0013778 21072170 PMC2970611

[B140] Weigle D. S. Duell P. B. Connor W. E. Steiner R. A. Soules M. R. Kuijper J. L. (1997). Effect of Fasting, Refeeding, and Dietary Fat Restriction on Plasma Leptin Levels1. J. Clin. Endocrinol. Metab. 82, 561–565. 10.1210/jcem.82.2.3757 9024254

[B141] Wilkinson M. J. Manoogian E. N. C. Zadourian A. Lo H. Fakhouri S. Shoghi A. (2020). Ten-Hour Time-Restricted Eating Reduces Weight, Blood Pressure, and Atherogenic Lipids in Patients with Metabolic Syndrome. Cel Metab. 31, 92–104. e105. 10.1016/j.cmet.2019.11.004 PMC6953486 31813824

[B142] Wu B. Sun X. Gupta H. B. Yuan B. Li J. Ge F. (2018). Adipose PD-L1 Modulates PD-1/pd-L1 Checkpoint Blockade Immunotherapy Efficacy in Breast Cancer. Oncoimmunology 7, e1500107. 10.1080/2162402X.2018.1500107 30393583 PMC6209395

[B143] Wu Q. Li J. Li Z. Sun S. Zhu S. Wang L. (2019). Exosomes from the Tumour-Adipocyte Interplay Stimulate Beige/brown Differentiation and Reprogram Metabolism in Stromal Adipocytes to Promote Tumour Progression. J. Exp. Clin. Cancer Res. 38, 223. 10.1186/s13046-019-1210-3 31138258 PMC6537177

[B144] Xiao Z. Dai Z. Locasale J. W. (2019). Metabolic Landscape of the Tumor Microenvironment at Single Cell Resolution. Nat. Commun. 10, 3763. 10.1038/s41467-019-11738-0 31434891 PMC6704063

[B145] Yang J. Yan J. Liu B. (2018). Targeting VEGF/VEGFR to Modulate Antitumor Immunity. Front. Immunol. 9, 978. 10.3389/fimmu.2018.00978 29774034 PMC5943566

[B146] Zhang W.-C. Qin F. Wang X.-J. Liu Z.-F. Zhu L. Zeng A. (2019). Adipose-Derived Stromal Cells Attenuate Adipose Inflammation in Obesity through Adipocyte Browning and Polarization of M2 Macrophages. Mediators Inflamm. 2019, 1–10. 10.1155/2019/1731540 PMC6913309 31871424

[B147] Zhao H. Shang Q. Pan Z. Bai Y. Li Z. Zhang H. (2018). Exosomes from Adipose-Derived Stem Cells Attenuate Adipose Inflammation and Obesity through Polarizing M2 Macrophages and Beiging in White Adipose Tissue. Diabetes 67, 235–247. 10.2337/db17-0356 29133512

[B148] Zhao X. Yang J. Huang R. Guo M. Zhou Y. Xu L. (2021). The Role and its Mechanism of Intermittent Fasting in Tumors: Friend or Foe? Cancer Biol. Med. 18, 63–73. 10.20892/j.issn.2095-3941.2020.0250 33628585 PMC7877171

[B149] Zhao Y. Butler E. B. Tan M. (2013). Targeting Cellular Metabolism to Improve Cancer Therapeutics. Cell Death Dis 4, e532. 10.1038/cddis.2013.60 23470539 PMC3613838

[B150] Zheng X. Zhang N. Qian L. Wang X. Fan P. Kuai J. (2020). CTLA4 Blockade Promotes Vessel Normalization in Breast Tumors via the Accumulation of Eosinophils. Int. J. Cancer 146, 1730–1740. 10.1002/ijc.32829 31840816

[B151] Zhou M. Zhao Y. Ding Y. Liu H. Liu Z. Fodstad O. (2010). Warburg Effect in Chemosensitivity: Targeting Lactate Dehydrogenase-A Re-sensitizes Taxol-Resistant Cancer Cells to Taxol. Mol. Cancer 9, 33. 10.1186/1476-4598-9-33 20144215 PMC2829492

[B152] Zwick R. K. Guerrero-Juarez C. F. Horsley V. Plikus M. V. (2018). Anatomical, Physiological, and Functional Diversity of Adipose Tissue. Cel Metab. 27, 68–83. 10.1016/j.cmet.2017.12.002 PMC6050204 29320711

